# Nanotherapeutics for Nose-to-Brain Drug Delivery: An Approach to Bypass the Blood Brain Barrier

**DOI:** 10.3390/pharmaceutics13122049

**Published:** 2021-11-30

**Authors:** David Lee, Tamara Minko

**Affiliations:** 1 Department of Pharmaceutics, Ernest Mario School of Pharmacy, Rutgers, The State University of New Jersey, 160 Frelinghuysen Road, Piscataway, NJ 08854, USA; ddlee1226@gmail.com; 2 Rutgers Cancer Institute of New Jersey, Rutgers, The State University of New Jersey, 195 Little Albany Street, New Brunswick, NJ 08903, USA; 3 Environmental and Occupational Health Science Institute, Rutgers, The State University of New Jersey, 170 Frelinghuysen Road, Piscataway, NJ 08854, USA

**Keywords:** nasal delivery, brain-targeting, neurogenerative disorders, CNS disorders, nanotechnology, nanomedicine

## Abstract

Treatment of neurodegenerative diseases or other central nervous system (CNS) disorders has always been a significant challenge. The nature of the blood-brain barrier (BBB) limits the penetration of therapeutic molecules to the brain after oral or parenteral administration, which, in combination with hepatic metabolism and drug elimination and inactivation during its journey in the systemic circulation, decreases the efficacy of the treatment, requires high drug doses and often induces adverse side effects. Nose-to-brain drug delivery allows the direct transport of therapeutic molecules by bypassing the BBB and increases drug concentration in the brain. The present review describes mechanisms of nose-to-brain drug delivery and discusses recent advances in this area with especial emphasis on nanotechnology-based approaches.

## 1. Introduction

The brain is one of the most complex, vital organs that accepts signals from sensory organs and regulates most body functions. It controls voluntary and involuntary movements, hormone secretion, memory encoding, and the functions of many other organs [1]. Since the brain has such a critical role in the human body, it is protected externally and internally. It is protected by a skull with different membrane layers that prevent external damage and is internally protected by cerebrospinal fluid (CSF), the CSF-blood barrier, and the blood-brain barrier (BBB). These barriers help maintain the homeostasis of the brain and prevent physical damage, infections, endotoxins, and any harmful effects [2,3].

The integrity of these protective mechanisms may be altered for various reasons such as trauma, mutation, aging and may lead to neurological disorders. As the brain regulates the whole body, the damage of this control center has a detrimental effect on both physical and mental health. According to the World Health Organization (WHO), death due to Alzheimer’s disease and other dementias more than doubled between 2000 and 2019, making it the 7th leading cause of death globally [4]. Not only dementia but also other neurological disorders, such as stroke, epilepsy, and Parkinson’s disease, are significant causes of hospitalization and mortality worldwide. In 2016, neurological disorders were the leading cause of disability-adjusted life-years (sum of years of life lost and years lived with disability) and the second leading cause of death [5]. Patients suffering from chronic neurological disorders may also end up facing depression and suicidal ideation [6].

Researchers have invented several different therapeutic agents to treat these devastating neurological disorders. Still, these agents are mainly used for slowing down the progression of disease and cannot completely reverse the condition [7]. One of the main reasons for this limited therapeutic effect is the presence of the BBB, which blocks more than 98% of neurotherapeutic molecules into the central nervous system (CNS) [8]. The BBB consists of endothelial cells of capillaries, astrocyte end-feet surrounding the outside of brain capillary endothelial cells, and pericytes embedded in the capillary basement membrane. The capillaries are non-fenestrated vessels with tight junctions, limiting the paracellular pathway of these therapeutic molecules [9]. Moreover, P-glycoprotein and other ATP-binding cassette transporters prevent the accumulation of the therapeutic drugs and pump those molecules out of the brain [10,11]. Due to the tight junctions that limit the paracellular route of drug delivery, the most possible pathways for drug transport to the CNS are the transcellular route (limiting the drug molecule to be highly lipophilic and molecular weight < 500 Da), receptor-mediated endocytosis, and carrier-mediated transport. However, in order to employ these mechanisms of drug entry into the brain, a drug or delivery vehicle needs to meet specific criteria for this to occur [12,13,14,15]. In addition, circumventricular organs with permeable endothelial cells of capillaries as well as specialized permeable zones of the brain potentially can also be used for the delivery of therapeutics to the brain tissues [16].

Many researchers proposed alternative strategies to overcome this physiological barrier, such as intracerebroventricular injection and intrathecal injection. Intracerebroventricular and intracerebral injection allow neurotherapeutic molecules to reach a high concentration in the brain. Still, the injection solution and drug itself need to meet many conditions, such as pH, volume, diluents, and preservatives in order to be injected directly into the brain structures, which creates certain difficulties for using these alternative routes for the brain delivery [17]. Intrathecal injection is more common, especially in oncology, to treat cancer in CNS, but its application in neurology is still limited. Deep brain stimulation involves the implantation of electrodes in the brain to treat Parkinson’s disease and has shown significant progress. However, this method is an invasive and costly technique [18]. Implantable drug reservoirs with prolonged drug release (e.g., GLIADEL^®^ Wafer) are also used for local brain delivery [19,20].

Since all of the procedures described above are invasive and costly, the intranasal delivery system has attracted attention as a route for potential drug delivery to the brain. Traditionally, intranasal delivery is used to promote local effects for the treatment of rhinitis or allergy. However, due to its many favorable characteristics, including non-invasiveness, good patient compliance, and ease of administration, intranasal delivery has also been used for systemic delivery. Its application has been increasing in the market, as it has shown efficacy in the flu vaccine, pain and migraine management, smoking cessation, and other areas [21]. Many studies have shown that intranasal delivery of small and large molecules can directly target the brain. Olfactory mucosa is the region that BBB does not protect, and it has direct contact with the brain. Also, it has the potential to decrease the accumulation of therapeutic molecules in the major organs, such as the liver, spleen, and kidney, and therefore reduce systemic side effects [22,23].

The present review is a comprehensive synopsis and analysis of the current landscape of nose-to-brain (N2B) delivery for nanotherapeutics from a wide range of perspectives including but not limited to mechanistic biology, transport kinetics, formulations, and clinical applications in both recent & historical context. Herein, the anatomy of the nose and prospective pathways of N2B delivery, challenges associated with those respective routes, basic pharmacokinetic parameters and expressions are discussed in greater detail. Furthermore, the application of various nanotherapeutic approaches for N2B delivery is assessed amongst neurological and other CNS disorders.

## 2. Nasal Cavity

To understand the different mechanisms of drug absorption through the nasal cavity to the brain, it is essential to know the anatomical and cellular structure of the nasal cavity.

### 2.1. Anatomy of the Nasal Cavity

**Figure 1:**
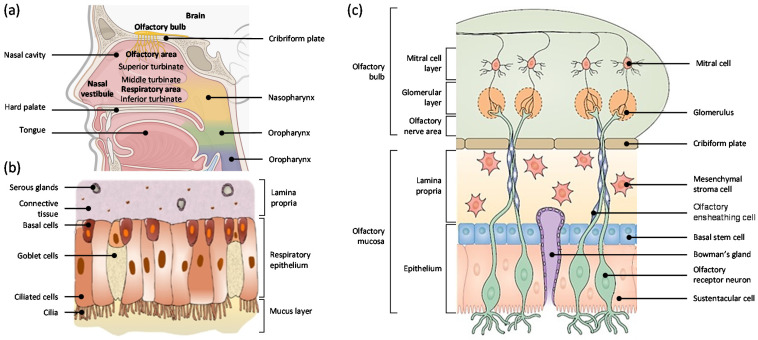
(**a**) Anatomy of the human nasal cavity. Squamous mucosa is located at the nasal vestibules. Respiratory mucosa is consisted of inferior, middle, and superior turbinate forming respiratory area. The olfactory mucosa is located underneath the cribriform plate in the olfactory area. (**b**) The respiratory mucosa. It is comprised of the lamina propria, respiratory epithelium, and a mucus layer. Within the respiratory epithelium, there are basal, goblet, and ciliated cells. Adapted with permission from [27], Elsevier, 2018. (**c**) The olfactory system consists of the olfactory mucosa, which is in the nasal cavity, and the olfactory bulbs, which are in the brain. The mucosa is composed of a pseudostratified epithelium containing olfactory receptor neurons (OSNs), Bowman’s glands, sustentacular cells, basal cells, and the lamina propria. OSNs have receptors that can entrap molecules and transmit information to glomeruli in the olfactory bulb. These neurons are ensheathed by glia, known as olfactory ensheathing cells (OECs). After damage or during normal cell turnover, newly formed OSNs are guided back by OECs into the olfactory bulb, where they re-synapse with glomeruli. Adapted with permission from [32], Springer Nature, 2020.

The nasal cavity extends around 12–14 cm in length, 5cm in height, has a total volume of 15–20mL, and a surface area of between 150 to 200 cm^2^ [24,25,26]. There are three kinds of turbinate: the superior, the middle, and the inferior turbinate, and they are responsible for humidifying, filtering, and warming the inspired air through nostrils [27,28]. The nasal cavity can be divided into three sections: the nasal vestibule, the respiratory section, and the olfactory section (Figure 1a) [28]. The nasal vestibule is located in the most anterior part of the nasal cavity, and it consists of hairs, sebaceous, and sweat glands [28,29]. The respiratory section is mainly dominated by the middle and the inferior turbinate, and it serves as a passage for air to the lungs. The olfactory area is located on the superior turbinate, covering about 10 cm^2^, and contains olfactory receptors, which are responsible for the sense of smell [28,30,31]. In terms of drug absorption through intranasal delivery, respiratory and olfactory mucosa are the main sites of interest.

### 2.2. Respiratory Mucosa

Respiratory mucosa consists of 80–90% of the total surface area in the human nasal cavity, and it is highly vascularized, making it a significant site for systemic drug absorption [25]. Respiratory mucosa consists of various cell types and glands, such as basal cells, goblet cells, ciliated epithelial cells, and serous glands (Figure 1b) [27,28]. Basal cells are progenitor cells that can differentiate into other cell types found within the epithelium and also help to attach ciliated and goblet cells to the basal lamina [33]. Goblet cells secrete mucus composed of mucin (high molecular weight glycoproteins), water, salts, a small group of proteins, and lipids [34]. Mucus forms a layer in the respiratory epithelium and serves as a first-line defense by entrapping any inhaled materials or irritants [35,36]. Ciliated cells help to remove this mucus towards the nasopharynx, which results in mucociliary clearance (MCC) [25,37]. Serous glands secrete watery fluid and other antimicrobial proteins, which serve as part of innate immunity [38].

### 2.3. Olfactory Mucosa

The olfactory mucosa is located on the top of the nasal cavity and takes up about 5~10% of the total surface area of the human nasal cavity [25]. The olfactory mucosa (Figure 1c) consists of olfactory receptor neurons, so-called olfactory sensory neurons (OSN), the olfactory epithelium, and the lamina propria [27,32]. The olfactory nerve is the first cranial nerve that transmits sensory information related to smell [39]. OSNs are non-myelinated neurons and located in the nasal epithelium. OSNs have direct contact with airborne substances through odorant chemoreceptors located in the apical surface of the olfactory mucosa, and each OSN expresses only one receptor [40]. Humans have approximately 400 olfactory receptors, whereas rodents have approximately 1000 olfactory receptors [41]. Each OSN forms thick axon bundles in the lamina propria, and these bundles become olfactory nerves. They innervate the cribriform plate and create synaptic connections with glomeruli of mitral and tufted cells in the olfactory bulb [40,42,43].

OSNs have direct contact with the environment, airborne irritants, and microbial agents, so these exogenous compounds may cause injury or cell death of OSN. To maintain its function, neurogenesis of OSNs occurs in the nasal epithelium to regenerate the neurons. A few studies have suggested that the life span of OSNs is between 30–60 days, and the systemic apoptosis of OSNs occurs to protect the brain from infections [28,40,44]. During the neuronal regeneration, there is a delay of tight junction formation, which causes some gap and allows some substance penetration [45].

Olfactory epithelium, just like respiratory epithelium, consists of ciliated columnar cells covered by a mucus layer. However, cilia in the olfactory epithelium are non-motile and longer than those in the respiratory epithelium [43]. In the olfactory epithelium, two types of basal cells account for neuronal regeneration: globose basal cells and horizontal basal cells. Globose basal cells are progenitor cells for OSNs, and they account for the homeostasis of normal tissue in olfactory mucosa [46,47]. Horizontal basal cells are multipotent progenitor cells in the olfactory epithelium for normal turnover and help its regeneration from acute injury [48]. Not only basal cells but also supporting cells are present in the olfactory epithelium. Sustentacular cells (SUS) are supporting cells that enclose the OSNs in the olfactory epithelium region. Their primary function is to stabilize the structural and ionic integrity of OSNs [49].

Lamina propria of olfactory mucosa consists of numerous cell types and structures such as Bowman’s glands (BG) and olfactory ensheathing cells (OEC) [50]. BGs innervate the olfactory epithelium and secrete a mucus layer in the olfactory system [51]. The exact composition of the olfactory mucus is still unknown, but a histological study showed that these glands are positive for periodic acid-Schiff staining, indicating the presence of neutral glycoproteins [28,52]. OECs are glial cells that enwrap non-myelinated bundles of OSN and help to promote the regeneration of OSNs [53].

## 3. Pathways for Nose-To-Brain Delivery

Drug transport through the olfactory mucosa has been studied to deliver therapeutic substances to the brain to treat CNS diseases. As described earlier, it has the significant advantage of bypassing BBB and reducing systemic exposure. The pathways for N2B delivery have not been fully understood, but many recent studies have suggested some major possible pathways. One way is the direct transport of drugs to the brain through neuronal pathways such as olfactory or trigeminal nerves. The other way is the indirect transport of drugs through the vasculature and lymphatic system, leading to the brain crossing BBB [54]. Drug absorption from nose to brain may not be limited by one single mechanism, but may involve several pathways.

### 3.1. Olfactory Pathway

**Figure 2:**
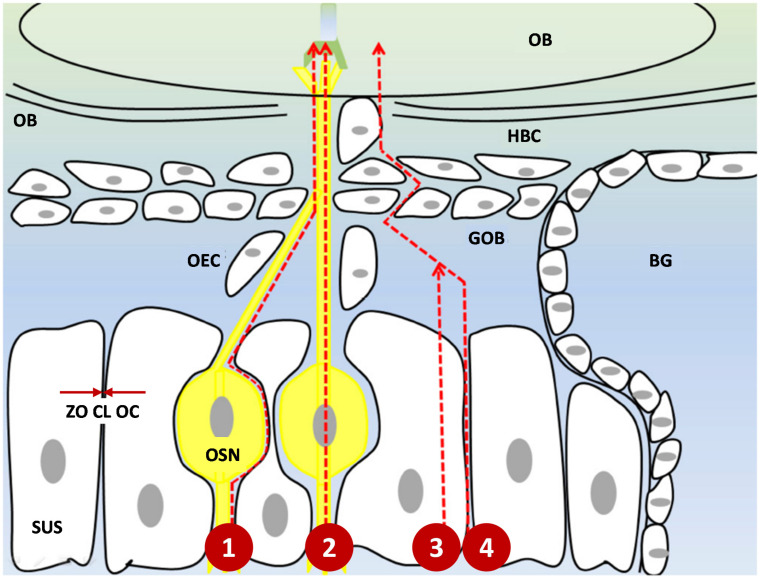
Four different routes of nose-to-brain drug delivery through olfactory mucosa. (1) Extra-neuronal pathway (2). Intra-neuronal pathway (3). Transcellular pathway (4). Paracellular pathway. The drug has to pass tight junctions (marked with red arrows) such as ZO, CL, and OC to travel through the intercellular space. N2B delivery is a mixture of these different pathways. Abbreviations: ZO: zonula occludens; CL: claudin; OC occludin; SUS: sustentacular cells; OSN: olfactory sensory neuron; OEC: olfactory ensheathing cell; GOB: globose basal cells; HBC: horizontal basal cells; BG: Bowman’s gland; CP: cribriform plate; OB: olfactory bulb. Modified from [28].

Major routes of drug transport from the olfactory pathway can be subdivided into four different categories: intra-and extra-neuronal pathways and paracellular and transcellular pathways (Figure 2) [23,27,42].

Olfactory neurons play a major role in the N2B delivery system. Therapeutic moieties can undergo endocytosis by OSN and form vesicles, leading to the intracellular axonal transport along the neurons, cross the cribriform plate, and to the olfactory bulb. Once they reach the brain, they will undergo exocytosis and will be distributed in the CNS [55]. The diameter of the human olfactory axon is between 0.1–0.7 µm, which makes it one of the smallest axons in the CNS [56]. This small diameter suggests that only small molecules within this range can be transferred through this intracellular axonal transport. Another limitation of intracellular axonal transport is the delayed-release. The mean speed of axonal transport is 25mm per day, which means that it may take hours and days for active moieties to be delivered to the brain [57]. Since many studies showed a rapid delivery of molecules through intranasal administration, it suggests that this pathway may not be the predominant one [45,58].

An extra-neuronal pathway of molecules occurs by crossing the gap between the OSN and the SUS in the epithelial layer. Then they reach the lamina propria, and are incorporated in the cleft between the axons and the OECs [25]. The active substances need to cross a tight epithelial junction to reach the cleft, but there is some gap due to the neuronal turnover in the olfactory epithelium, which allows the drug transport to occur, even for larger moieties [40,45].

A paracellular pathway occurs by crossing the olfactory epithelium through the gap along the SUS and crossing the basement membrane. Instead of incorporating in the cleft, the therapeutic molecules can reach the subarachnoid space and get delivered to the brain by crossing the blood-CSF barrier. This route does not require drugs to bind to receptors, and it is particularly suitable for hydrophilic and small molecules [30,59]. A transcellular pathway occurs by receptor-mediated endocytosis or passive diffusion of inhaled molecules through the membrane of the SUS [60]. This pathway is suitable for hydrophobic molecules.

### 3.2. Trigeminal Pathway

A trigeminal nerve is the fifth cranial nerve and is the largest cranial nerve which innervates both the olfactory and the respiratory mucosa. It has three different branches, consisting of the ophthalmic, maxillary, and mandibular nerves, and is responsible for delivering sensory and motor information of these areas to the spinal cord, the medulla, and the pons [27,58,61]. Among those branches, the ophthalmic and maxillary branches are involved for N2B delivery. Ophthalmic branches pass through the dorsal nasal mucosa and anterior part of the nose, and maxillary branches through the lateral wall of nasal mucosa [23,30]. Similar to the olfactory nerve pathway, drug transport via the trigeminal nerve occurs by multiple pathways. Once drug moieties reach the branches of the trigeminal nerve, they will merge at the trigeminal ganglion and enter the brain near the pons. Also, some portions of the trigeminal nerve are present near olfactory bulbs, so drug molecules can cross the cribriform plate and reach both the caudal and rostral areas of the brain [23,62].

### 3.3. Systemic Pathway

Drug transport of inhaled substances to the brain can occur indirectly through the respiratory epithelium via systemic circulation and the lymphatic system. Since the respiratory epithelium is highly vascularized with a combination of a continuous and fenestrated endothelium, it gives access to blood circulation. However, these substances need to cross the BBB to reach the CNS, which is the rate-limiting step. The systemic pathway mainly occurs for the small and lipophilic substances so that they can cross the BBB transcellularly [27,30,58].

## 4. Potential Challenges for Nose-To-Brain Delivery

Nose-to-brain delivery has many advantages, including bypassing BBB, less systematic side effects, and increasing patient compliance using a non-invasive approach. However, there are a few challenges, such as optimizing mucus penetration and mucociliary clearance (MCC).

### 4.1. Cilia and Nasal Mucus Transport

There are motile and non-motile cilia in the nasal cavity. Motile cilia are mostly present in the respiratory epithelium, whereas non-motile cilia are prevalent in the olfactory epithelium. Motile cilia have a motor protein called dynein, which generates motion, and non-motile cilia play roles in sensory function and transportation [28,63]. A small portion of respiratory mucosa is present in the olfactory region, allowing mucus transport in the olfactory region [64].

Nasal mucus consists of about 95% water, 2~3% mucin, 1% salts, and other cellular debris such as DNA, albumin, immunoglobulins, and lipids [65]. As described earlier, mucus is secreted by goblet cells and Bowman’s glands in the respiratory and olfactory epithelium, respectively, at the rate of 1.5~2 L daily. It is known to have antimicrobial and humidifying effects as wells as providing surface electrical activity. Mucus has a mesh-like structure that allows the penetration of particles less than 1 µm in diameter [66]. Therefore, therapeutic moieties need to be small enough to penetrate the mucus. Mucus can also perform interaction filtering, regardless of the size of particles. These interactions include electrostatic forces, hydrophobic, and Van der Waal’s bonds [67]. Due to these interactions, lipophilic drugs have more difficulty penetrating the mucus layer than hydrophilic drugs.

### 4.2. Mucociliary Clearance

Mucociliary clearance (MCC) is an interaction between the cilia and mucus layers, which helps inhaled toxic substances to adhere and transport toward the nasopharynx and gastrointestinal tract [68,69]. There is an inter-individual difference in MCC, but the speed is estimated to be 6 mm/min on average. MCC is one of the major factors to consider for N2B delivery since it can affect drug bioavailability. Drug formulation should be able to stay long enough to penetrate the mucus and adhere to the local nasal epithelium before being washed away. Once the inhaled molecules cross the mucus, they have good permeability to the nasal epithelium [70].

MCC can vary based on environmental and pathological factors. Decreased mucus viscosity, increased mucus production, and increased ciliary beat frequency will increase MCC. In contrast, the inhalation of sulfur dioxide, smoking, reduced temperature in the nasal cavity, and thickened mucus will decrease MCC. Asthma, rhinitis, allergy, and sinusitis can change MCC by affecting ciliary beat frequency or mucus production [69,71]. MCC can also be influenced by drugs that affect ciliary beat frequency. Anesthetics, cholinergic inhibitors, alpha-adrenergic receptor agonists, corticosteroids, and anti-histamine drugs inhibit MCC, whereas beta-adrenergic agonists and cholinergic agonists increase the ciliary beat frequency and stimulate MCC [70]. Therefore, N2B delivery may have variable bioavailability in the brain, depending on patients’ physiological conditions and medications.

## 5. Pharmacokinetics of Nose-To-Brain Delivery

Drug absorption through N2B delivery, as distinct from a conventional pathway for brain delivery (oral, parenteral, and transdermal), requires specific pharmacokinetic indexes to measure its effectiveness.

Drug targeting efficiency (DTE) is a parameter that represents the efficiency of the drug to reach the brain via the intranasal route relative to that obtained via the systemic route (1). AUC is the area under the curve representing drug concentration over time for the duration of the study [72]. Values can range from 0 to +∞, and the values above 100% indicate better efficient brain targeting through IN than IV, whereas values below 100% represent the opposite.

(1) $$\mathrm{DTE}{\left(\%\right)}_{\left(\mathrm{IN}\right)}=\frac{{\left(\frac{{\mathrm{AUC}}_{\mathrm{Brain}}}{{\mathrm{AUC}}_{\mathrm{Blood}}}\right)}_{\mathrm{IN}}}{{\left(\frac{{\mathrm{AUC}}_{\mathrm{Brain}}}{{\mathrm{AUC}}_{\mathrm{Blood}}}\right)}_{\mathrm{IV}}}\times 100$$

DTE does not describe which pathway contributed to the drug concentration in the brain. Instead, it implies that intranasal administration leads to higher brain bioavailability than intravenous administration.

To calculate whether intranasal delivery directly leads drugs to reach the brain or not, we can use direct transport percentage (DTP). DTP is a percentage of the dose reach to the brain via IN compared to the overall delivery of the drug to the brain (2). It represents the drug fraction from direct transport to the brain.

(2) $$\mathrm{DTP}{\left(\%\right)}_{\left(\mathrm{IN}\right)}=\frac{{\mathrm{AUC}}_{\mathrm{BrainIN}}-F}{{\mathrm{AUC}}_{\mathrm{BrainIN}}}\times 100$$

F is the brain AUC fraction from the systemic circulation (indirect pathway) (3).

(3) $$F=\frac{{\mathrm{AUC}}_{\mathrm{BrainIV}}}{{\mathrm{AUC}}_{\mathrm{BloodIV}}}\times {\mathrm{AUC}}_{\mathrm{BloodIN}}$$

The values of DTP can range from -∞ to 100%. A positive DTP value indicates a contribution of the direct N2B pathway to the drug levels, whereas 0 or negative values indicate the drug prefers to be delivered to the brain through systemic circulation after IV administration. These quantitative data help build advanced PK-PD models to predict CNS concentration for N2B delivery [73]. One limitation of DTE and DTP is that poorly permeable drugs to BBB will lead to high values, so it does not always correlate to high bioavailability in the brain.

B%_Brain IN/IV_ is used to measure the drug accumulation in the brain from IN compared to that of IV (4). Values above 100% indicate a better brain drug accumulation through IN administration.

(4) $${B\%}_{\mathrm{Brain}\;\mathrm{IN}/\mathrm{IV}}=\frac{{\mathrm{AUC}}_{\mathrm{BrainIN}}}{{\mathrm{AUC}}_{\mathrm{BrainIV}}}\times 100$$

Relative bioavailability_Brain_ is a measure of brain drug accumulation with nanosystem IN compared to drug solution IN (5). Since many N2B delivery systems use nanocarriers to deliver drugs, it may be necessary to calculate the effectiveness of the nanosystem compared to that of free drug solution.

(5) $${\mathrm{Relative}\;\mathrm{bioavailability}}_{\mathrm{Brain}}=\frac{{\left({\mathrm{AUC}}_{\mathrm{BrainIN}}\right)}_{\mathrm{nanosystem}}}{{\left({\mathrm{AUC}}_{\mathrm{BrainIN}}\right)}_{\mathrm{solution}}}\times 100$$

Values above 100 will indicate a better drug accumulation with the nanosystem than the drug solution. Using this relative bioavailability concept, we can also compare the relative DTE and DTP of the nanosystem and drug solution using the following equations:

(6) $$\mathrm{RDTE}\%=\frac{{\mathrm{DTE}\%}_{\mathrm{INnanosystem}}}{{\mathrm{DTE}\%}_{\mathrm{INsolution}}}\times 100$$

(7) $$\mathrm{RDTP}\%=\frac{{\mathrm{DTP}\%}_{\mathrm{INnanosystem}}}{{\mathrm{DTP}\%}_{\mathrm{INsolution}}}\times 100$$

## 6. The Potential Role of Nanotechnology for Nose-To-Brain Delivery

Pharmaceutical nanotechnology has been widely used to deliver therapeutic molecules to the targeted area. The size of the particles is in the nano range (1–1000 nm), and these particles typically form a colloidal dispersion [74,75]. The use of nanotechnology in N2B delivery is very promising. It can increase the residence time of the drug at the site of absorption, promote its mucosal permeation and cellular internalization, increase drug solubility, control the release of the encapsulated drug, and reduce systemic side effects by decreasing the drug distribution to the non-targeted area. All these characteristics favor the use of nanoparticles (NPs) for N2B delivery [76].

Although nanotechnology has been widely used in drug delivery for its favorable characteristics, the effect and accumulation in the human body should not be neglected. Once nanocarriers enter the biological system, proteins, lipids, and other biological molecules in the body will be adsorbed on the surface of nanocarriers and form the so-called “biocorona” [77]. The biocorona can alter physicochemical properties such as size, shape, and hydrophilicity of original nanocarriers through nanoparticle-biomolecule interactions [78]. Also, the pharmacokinetic profile, such as cellular uptake, half-life, and distribution can be modified [79,80]. The biocorona can be recognized by complement receptors on macrophages and undergo increased cellular uptake and accumulated in the liver and spleen [81]. Some studies showed that metal-based nanoparticles may cause negative effects on the cardiovascular system and the nervous system. Increased inflammatory cytokines, arrhythmia, as well as increased oxidative stress and neurotoxicity could occur after the administration of titanium dioxide and silica nanoparticles, which are a commonly used nano-formulation in the industry [82,83]. Since peptides and lipids are present in the nasal mucus, there is a high chance that the inhaled nanoparticles will form the biocorona and may alter their physicochemical properties and cellular uptake. Therefore, the characteristics of the biocorona need thorough evaluations to effectively translate preclinical data to a safer and more efficient nanosystem for clinical application.

Many different types of NPs have been used for N2B delivery, but the two most used types of nanoparticle carriers will be discussed in this review: lipid-based NPs and polymer-based NPs [84]. These nanoparticle carriers help to increase drug accumulation in the brain by increasing stability, solubility, and mucoadherence.

### 6.1. Lipid-Based Nanoparticles

**Figure 3:**
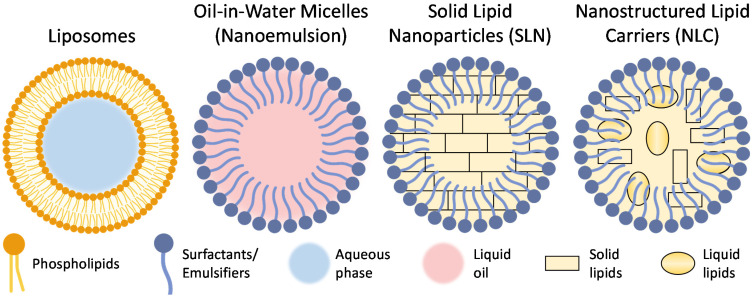
Most widely used lipid-based nanoparticles.

Lipid-based nanoparticles have been widely investigated for drug delivery systems. These NPs are amphiphilic, being able to transfer both hydrophilic and hydrophobic materials in one particle [85]. Lipid-based carriers are made from biocompatible, biodegradable lipids similar to those consisting of the cell membrane. These features allow them to penetrate the cells efficiently and limit their toxicity. Most commonly used lipid-based NP formulations are (Figure 3) liposomes, nanoemulsions formed with micelles, solid lipid nanoparticles (SLN), and nanostructured lipid carriers (NLC) [86,87]. These lipid-based NPs are often modified with polymers such as polyethylene glycol (PEG) or poloxamers. PEG is a hydrophilic polymer that is biocompatible and stabilizes NPs [88]. Furthermore, it acts as a mucus penetration enhancer by decreasing interaction with mucin [76]. Poloxamers, similar to PEG, are water-soluble, non-ionic surfactants and consist of a triblock copolymer of hydrophobic polypropylene glycol and two hydrophilic blocks of PEG. They have low toxicity, good drug release, and are compatible with many different chemicals, making them useful tools for drug delivery [89]. Poloxamer 407 (Pluronic F127) and 188 (Pluronic F-68) both have high contents of PEG (70% and 80%, respectively) and can help decrease mucus viscosity and increase penetration by interacting with lipid membranes and tight junctions [90,91].

It is important that lipid-based NPs can cross the epithelial and respiratory epithelium transcellularly and penetrate to the brain, which makes them an attractive option for N2B delivery. Moreover, lipid-based NPs can be indirectly absorbed into the systemic circulation, and have a good chance of crossing the BBB because of their lipophilic nature [92]. Lastly, medications that target the brain are relatively hydrophobic, which makes lipid-based NP attractive delivery vehicles that can increase drug solubility and bioavailability in the brain [93].

#### 6.1.1. Liposomes

Liposomes are one of the most widely used lipid-based NPs for drug delivery systems. Typically, a liposome has a single or several phospholipid bilayers, often with other lipids such as cholesterol or phosphatidylcholine. Using various types of lipids, the physical characteristics of liposome membranes may vary in terms of size and surface charge. For instance, neutral or slightly negatively charged liposomes can incorporate both hydrophilic (inside their aqueous core) or hydrophobic (inside the lipid membrane) active ingredients. In contrast, the positively charged liposomes can form multiplexes with negatively charged nucleic acid [94,95,96,97,98].

Many studies of N2B delivery have used a liposome as a nanocarrier to treat different types of CNS disorders [99,100,101,102,103,104]. Al Asmari et al. formulated a donepezil-loaded liposome using 1,2-distearoyl-sn-glycero-3-phosphocholine (DSPC), cholesterol, and PEG to evaluate the brain and plasma pharmacokinetics after intranasal administration [99]. Donepezil is a cholinesterase inhibitor, and it is a commonly used medication to treat Alzheimer’s disease. In their study, the size of nanoparticles was 102 ± 3.3 nm, the surface charge was −28.31 ± 0.85 mV, the polydispersity index (PDI) was 0.28 ± 0.03, and drug encapsulation efficiency (EE) was 84.91 ± 3.31%. The drug release from the liposomes had biphasic characteristics: an initial rapid release phase for 2 h followed by a sustained release up to 8 h. The AUC of donepezil liposome through intranasal (IN) delivery was higher than the AUC of oral (PO) and IN of free donepezil. The bioavailability of donepezil delivered by liposomes via the IN route in the brain was two times higher than that of free IN donepezil (*p* < 0.05) but showed no significant difference in terms of half-life. The histopathological study showed no evident signs of injury in major organs such as the liver, lung, heart, spleen, kidney, brain, and olfactory bulb after nasal administration of the liposomal formulation of donepezil in rats. This study showed the promising role of liposome as a carrier for improving the bioavailability of donepezil to the brain with N2B delivery systems [99]. Hoekman and et al. developed a fentanyl-loaded liposome with an arginine-glycine-aspartate (RGD) peptide and underwent aerosolization for intranasal delivery [101]. Rats treated with RGD-liposome IN had a higher analgesic effect than those with free fentanyl IN (AUC 1387.1 vs. 760.1%) and 20% reduced plasma drug exposure (AUC_0–120_ 208.2 vs. 284.8 ng·min/mL). The RGD peptide liposomes bind to integrin proteins on the nasal epithelium and eventually increase the retention of fentanyl in the nasal and olfactory epithelium [105]. In addition, the liposomes worked as a drug reservoir, as there was a significant increase in the overall analgesic effect without affecting the onset of action, but lasted six times longer than the free fentanyl solution. Intranasal liposomal delivery potentially showed increased drug concentration in the brain as well as a decreased systemic exposure [101].

#### 6.1.2. Solid Lipid Nanoparticles

Solid lipid nanoparticles (SLNs) are the newer generation of lipid-based nanocarriers, which are lipid emulsions where a solid lipid has replaced the liquid lipid. They are usually 100–300 nm in diameter and form a solid lipid matrix. They are often comprised of physiological lipids in water or aqueous surfactants [106]. SLNs have several advantages for drug delivery: they can be produced without using organic solvents, have high physical stability, and enhanced, controlled release of loaded drugs. The major drawbacks of SLNs include limited drug loading efficiency (especially for hydrophilic molecules) due to inflexibility of their shape, and undesired particle growth by agglomeration, which may lead to the burst release of the drug [107,108,109,110].

Patel et al. formulated SLNs that incorporates risperidone, an atypical antipsychotic agent, to increase its bioavailability and biodistribution [111]. Compritol 888 ATO was used for the lipid and Pluronic F-127 for the surfactant components of SLNs. The concentration of the radiolabeled risperidone was about three times higher in the risperidone SLNs delivered via IN group than the risperidone IV group and marginally higher than the risperidone SLN IV group. The concentration of the risperidone in the blood from SLN IN was twice as low as that from IV SLN, which can potentially enhance drug specific activity and lower systematic side effects [111].

#### 6.1.3. Nanostructured Lipid Carriers

Nanostructured lipid carriers (NLCs) represent a relatively recent generation of lipid-based NPs that are developed to overcome the disadvantages of SLNs. NLCs have a mixture of solid and liquid lipids, leading to higher drug loading and the prevention of the drug’s burst release [106]. NLCs are typically formulated by the double emulsion technique (*w/o/w*) and high-pressure homogenization [112]. Hydrophobic molecules have a higher solubility in liquid lipid than solid lipid, so higher encapsulation efficiency can be achieved [113]. Some limitations of NLC include decreased encapsulation efficiency for a combination of two or more therapeutic agents and relatively low drug loading capacity for hydrophilic drugs [114]. Madane et al. formulated curcumin-loaded NLC to increase brain bioavailability for brain cancer treatment. The study used precirol as a solid lipid, capmul MCM as a liquid lipid, Tween 80 as a surfactant, and soya lecithin as a stabilizer. NLCs were prepared using a high-pressure homogenization technique. The average particle size was 146.8 nm, with zeta potential (ZP) of −21.4 ± 1.87 mV, PDI of 0.18, and good entrapment efficiency (90.86%). The curcumin-NLC had a biphasic release pattern, with burst release at the initial stage followed by sustained release. Cmax of curcumin-NLCs after the IN administration was about 1.5 times higher than that of curcumin suspension IN. The relative bioavailability of curcumin-NLC IN was 439 ± 9.86% when compared with curcumin suspension IN. This study showed that the NLC could be potentially used for N2B delivery to treat CNS disorders [115].

#### 6.1.4. Nanoemulsions

Nanoemulsions are nano-sized colloidal systems comprised of micelles containing an oily phase, emulsifier, and aqueous phase. There are three types of nanoemulsions: oil in water, water in oil (so-called “reversed” micelles), and bi-continuous nanoemulsion (interdispersed water and oil domain) [116,117]. Nanoemulsions can improve the bioavailability and stability of the drug, especially lipophilic drugs, and provide higher drug absorption with a greater surface area from nano-sized droplets [118]. However, it is thermodynamically unstable and can lead to poor stability and the release of the encapsulated molecules during storage [119]. Iqbal et al. used a nanoemulsion (NE) to encapsulate letrozole (LZ), an aromatase inhibitor [120]. LZ is clinically indicated for breast cancer, but letrozole has been recently studied to reduce epilepsy [121,122]. The NE was prepared using Triacetin for the oil phase, Tween 80 for surfactant, and PEG 400 for co-surfactant. The LZ-NE had a mean diameter of 95.59 ± 2.34 nm, a PDI of 0.162 ± 0.012, and a ZP of −7.12 ± 0.12 mV. LZ-NE IN significantly increased the latency to seizure, decreased the number of seizures and a percent of seizure occurrence in kainic acid-induced status epilepticus mice compared to letrozole solution administered intraperitoneally. Although the study used different routes of administration for letrozole solution for comparison, it showed some neuroprotective effect by decreasing 17β-estradiol, an enzyme that has neuronal excitability and seizure enhancing activity [120].

### 6.2. Polymer-Based Nanoparticles

Polymeric nanocarriers, either natural or synthetic polymers, have been used for N2B delivery to increase stability, control the drug release pattern and modify the surface of nanoparticles.

#### 6.2.1. Natural Polymer-Based Nanoparticles

Chitosan (CS) has been widely used for preparing different nanoparticles. Chitosan is a polysaccharide of D-glucosamine, and N-acetyl-D-glucosamine obtained from the deacetylation of chitin, which is the building material of insects and crustaceans [123]. The pKa value of chitosan is around 6.5, so it becomes protonated in acidic pH. The pH of mucus is between 5.5–6.5, which makes chitosan positively charged and increases its stability [124,125]. Since both the olfactory and respiratory epithelium are negatively charged, chitosan-based NPs stay longer in the olfactory and respiratory mucosa and increase the bioavailability of the encapsulated drug for the brain. Also, it acts as a permeation enhancer that helps open the tight junctions between epithelial cells and allows the paracellular transport of materials. It can translocate proteins that consist of the tight junction, ZO-1, and CLs, from the cell membrane to the cytosol by modulating protein kinase C [126,127]. Chitosan-based nanoparticles are degraded by different enzymes such as chitosanase, cellulases, pepsin and lipases [128].

Even though chitosan has very promising characteristics for N2B delivery, there are some limitations of this material. Chitosan is insoluble under physiological pH and positively charged only in an acidic environment, which may interfere with bioadhesion [76]. Due to these limitations, many researchers have modified chitosan derivatives for N2B delivery. One example is trimethylchitosan (TMC), which has a better water solubility than naïve chitosan, and high positive charge under physiological pH [129,130]. Kumar et al. formulated TMC nanoparticles and loaded leucine-enkephaline, an analgesic neurotransmitter, for pain management. The permeability and brain accumulation of leucine-enkephalin TMC NPs IN were significantly higher than leucine-enkephalin solution IN [130].

Another modification of chitosan, thiolated-chitosan (TC), can increase mucoadhesion by forming disulfide bonds between the thiol group and mucus glycoproteins [131,132]. Singh et al. formulated selegiline-loaded TC NPs for the treatment of depression. The concentration of selegiline in the brain was significantly higher in TC NPs IN than the selegiline solution IN and unmodified chitosan-coated NPs IN. Behavior assessment in mice with TC NPs showed a more favorable response than unmodified chitosan-coated NPs in an immobility stress evaluation and sucrose preference test. Also, TC NPs successfully decreased oxidative stress and repleted the mitochondrial complex activity [131]. These results show that TC could be a valuable option for N2B delivery.

Alginic acid is a natural polysaccharide that is present in the cell walls of brown algae. The salt form, sodium, or calcium alginate are the primary forms that are currently used for drug delivery. It is hydrophilic and becomes viscous, and easily forms a gel when hydrated, which helps design controlled drug release [133].

Haque et al. loaded venlafaxine, a serotonin-norepinephrine reuptake inhibitor, into alginate NPs for the treatment of depression. The particle size was 173.7 ± 2.5 nm with ZP of +37.4 ± 1.74 mV, PDI of 0.391 ± 0.045, and EE of 81.3 ± 1.9%. Behavioral tests, such as forced swimming and locomotor activity tests, were measured in depressed rodents. The rodents treated IN with venlafaxine alginate NPs had similar behavioral tests results compared to non-depressed rodents and better results than venlafaxine solution and tablet groups. The drug targeting efficiency (DTE) and drug transport percentage (DTP) from venlafaxine alginate NPs IN were higher than those from venlafaxine solution IN (425.77% vs. 268.38% and 76.52% vs. 62.76%, respectively) [134]. It showed that alginate could be a useful carrier for N2B delivery.

#### 6.2.2. Synthetic Polymer-Based Nanoparticles

Synthetic polymers have been extensively used for formulation of various drug delivery systems. Many of these polymers are biodegradable and biocompatible, which makes them favorable carriers for N2B delivery. The most widely used polymers include poly(L-lactide-co-glycolide) (PLGA), poly(lactic acid) (PLA), and poly(glycolic acid) (PGA). They are used to prevent the degradation of drugs in the nasal cavity and promote hydrophobic drug loading due to their hydrophobicity [135,136]. These polymer-based nanoparticles encapsulate drugs through single or double emulsion techniques [137]. Like lipid-based liposomes, polymeric nanoparticles often undergo surface modification with PEG or poloxamers to increase stability, drug loading, and penetration through the nasal mucus [138].

**Figure 4:**
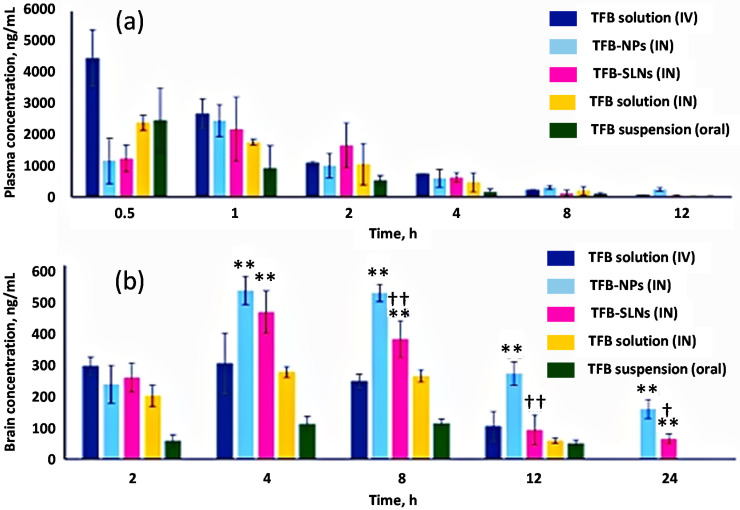
Plasma concentrations (**a**) and brain concentrations (**b**) of tarenflurbil (TFB). Values are expressed as mean ± SD (*n* = 4). ** *p* < 0.0001 when compared with TFB administered by IV solution, IN solution and oral suspension, ^††^ *p* < 0.0001 when compared with TFB-NPs and ^†^ *p* < 0.01 when compared with TFB-NPs. Adapted with permission from [139], Elsevier, 2016.

Muntimadugu et al. formulated tarenflurbil-loaded solid lipid nanoparticles (SLN) and PLGA NPs for effective brain penetration. Tarenflurbil (TFB) is a β-amyloid-42 peptide lowering agent and modulates γ-secretase, an enzyme responsible for β-amyloid plaque formation. TFB PLGA NPs used in one study had a size of 133.13 ± 7.82 nm, ZP of −30.25 ± 2.11 mV, PDI of 0.21 ± 0.02, and encapsulation efficiency of 64.11 ± 2.21% [139]. TFB SLN had a size of 169.87 ± 10.98 nm, ZP of −23.13 ± 2.32 mV, PDI of 0.24 ± 0.04, and EE of 57.81 ± 5.32%. Both formulations showed a biphasic release pattern: ~55% of TFB was released from both formulations in 2 h with a sustained release for 48 h. Higher absolute bioavailability, DTE, and DTP were recorded in TFB PLGA NPs than those in TFB SLN. The effectiveness of PLGA NPs was markedly observed in 8 h and 24-h post-administration as the concentration of TFB in the brain was significantly higher in the PLGA NPs group than the SLN group (*p* < 0.0001 and *p* < 0.01, respectively) (Figure 4). Both formulations yielded significantly higher drug concentration in the brain than IV and PO tarenflurbil, with no significant drug concentration difference in the spleen and heart [139]. This study implied that tarenflurbil could be a more suitable substrate for polymeric NPs than lipid-based NPs for N2B delivery.

## 7. Physicochemical Properties That Can Affect Nose-To-Brain Delivery

### 7.1. Particle Size

Particle size is one of the most crucial factors in the N2B delivery system. As stated above, the diameter of the OSN is between 0.1–0.7 µm, which limits the particle size to the nano range [56]. Also, smaller particles have less resistance to the mucous membrane penetration, as mucus forms a mesh-like structure. There are several studies that show that particle size can be a limiting factor for N2B delivery. Mistry et al. formulated chitosan or polysorbate 80 coated polystyrene NPs with a 100 and 200 nm particle size [140]. The study showed that nonmodified polystyrene NPs and polysorbate 80 coated NPs with the particle size of 100 nm were more suitable for olfactory epithelial cells than those with 200 nm diameter. However, none of the formulations were found in the olfactory bulb. Based on this study, the authors concluded that the optimal nanoparticle diameter for axonal transport is less than 100 nm [140].

**Figure 5:**
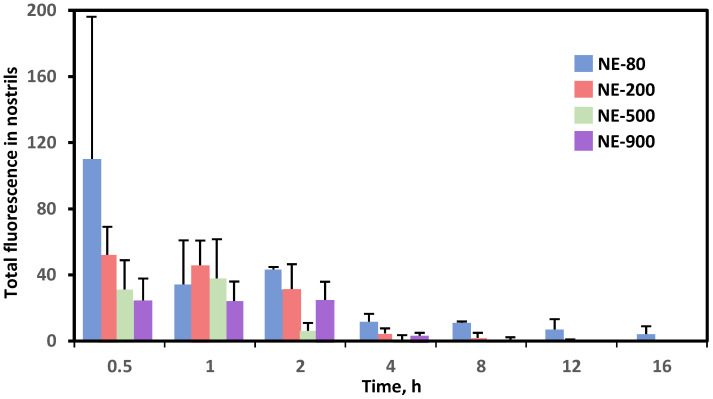
Quantification of total fluorescence of nanoemulsions (NE) with different sizes (80, 200, 500, 900 nm) in nostrils. Replotted using data from [141].

Similarly, Ahmad et al. formulated nanoemulsions (NE) of different sizes with various fluorescent markers and studied their biodistributions after intranasal administration [141]. The fluorescent images showed that regardless of the presence of chitosan, NEs with a particle size smaller than 100 nm have prolonged residence time in the nasal epithelium and slower MCC when compared with larger nanoparticles (200, 500, and 900 nm) (Figure 5). Moreover, NEs of 100 nm were able to be transported through both trigeminal and olfactory pathways, which can potentially increase the drug concentration in the brain [141].

### 7.2. Surface Charge

The nasal mucosa membranes are negatively charged in general, so positively charged particles are more likely to interact with nasal mucosa through electrostatic force [142]. This will lead to increased residence time and bioadhesion to the nasal epithelium. Due to this characteristic, many researchers have used positively charged carriers such as chitosan and its derivatives to increase drug bioavailability for N2B delivery. Mistry et al. showed that chitosan-coated carriers caused nanoparticles to interact with extracellular mucus for an extended period of time, allowing nanoparticles to cross the nasal epithelium paracellularly [140]. In a study by Gabal et al., cationic nanostructured lipid carriers (NLCs) with similar absolute ZP (+33 mV) had higher absolute bioavailability than anionic NLCs after intranasal administration (77.3% vs. 44%), but slightly lower drug targeting efficiency (128.6% vs. 158.5%) [143].

**Figure 6:**
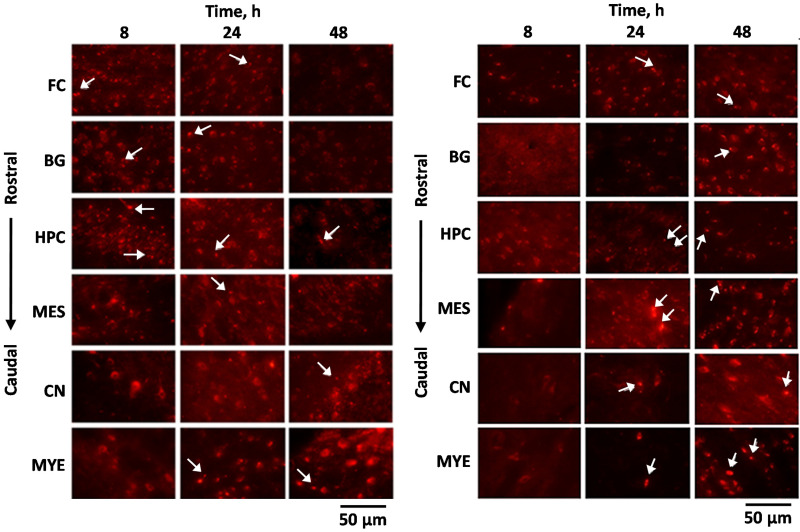
Localization of the negatively charged NPs (**left** panel) and positively charged NPs (**right** panel) after IN administration. Frontal cortex (FC); basal ganglia (BG); hippocampus (HPC); mesencephalon (MES); cerebellar nuclei (CN); myelencephalon (MYE). The white arrows indicate the presence of rhodamine-labeled NPs. Adapted with permission from [144], Elsevier, 2017.

Not only can the charge of NPs increase the residence time, but it can also impact its delivery pathway. Bonaccorso et al. formulated rhodamine B labeled polymeric nanoparticles with an opposite surface charge to evaluate the bioavailability after intranasal administration in mice. The study used poly-lactide-co-glycolic acid (PLGA) NPs to make negatively charged nanoparticles, and used chitosan to make them positively charged. The mean size of both types of NPs was smaller than 250 nm. The negatively charged NPs arrived at the rostral subregions after 8 h of IN administration and were further transported to the caudal region in 24 h. However, positively charged NPs arrived in caudal sub-regions after 24 h of IN administration and were transported to the rostral area (Figure 6). Since the fluorescent signal from negatively charged NPs appeared in early time points, it is suggested that they were delivered via the olfactory pathway with both intra and extra-neuronal pathways. On the other hand, positively charged NPs transported through the trigeminal nerve as the fluorescent signal was strong after 48 h in the posterior brain. The author suggested that the surface charge influences the delivery pathway and the time to reach the brain [144].

### 7.3. Lipophilicity

**Figure 7:**
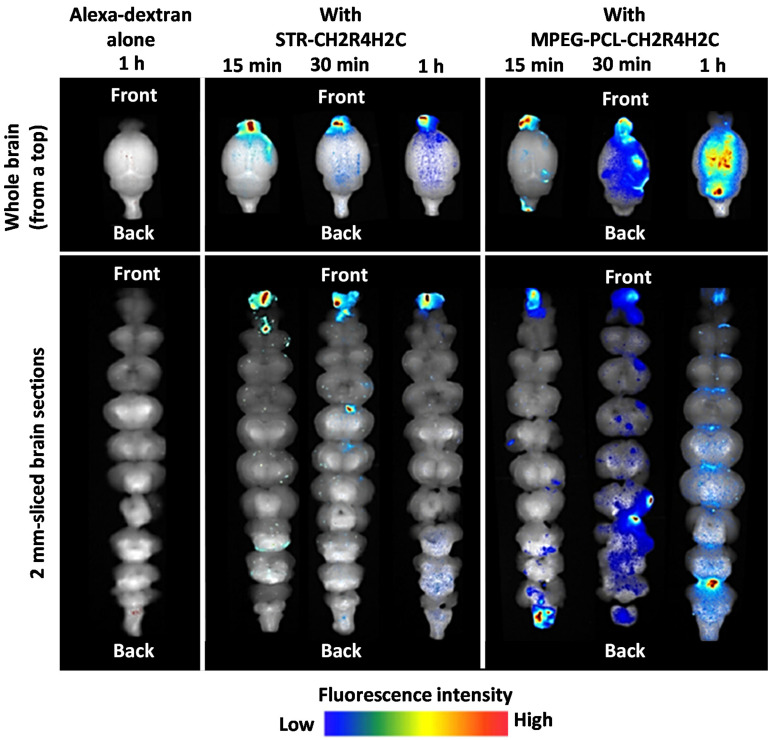
Dynamics of Alexa-dextran in brain tissue following IN administration of Alexa dextran alone, hydrophobic (STR-CH2R4H2C) and hydrophilic (MPEG-PCL-CH2R4H2C) in whole brain and 2 mm-sliced brain sections. Adapted with permission from [146], Elsevier, 2017.

Hydrophobic carriers are more likely to increase mucoadhesion as they form hydrophobic bonding with mucin’s hydrophobic domains and thus increasing the residence time. However, if the carrier is too hydrophobic, it will not penetrate the mucus due to hydrophobic interaction with mucin and will be cleared by MCC [145]. Therefore, it would be essential to have a fine balance between increasing the residence time and mucus penetration. Not only can hydrophobicity affect mucoadhesion, but it also may determine the pathway of N2B delivery and distribution in the brain, just like the charge of the nanoparticle. Kanazawa et al. designed an arginine-based peptide and conjugated it either with hydrophobic stearate (STR) or hydrophilic PEG-PCL copolymer to obtain stable micellar formulations. The study used Alexa-dextran for fluorescent imaging. The fluorescent images showed that both formulations had higher fluorescent activity in the nasal mucosa and the brain than the control (Alexa-dextran) (Figure 7). The nanoparticle complexed with hydrophilic peptide had higher intensity in the trigeminal nerve and more fluorescence spreading, which implies that it was transferred via multiple pathways. Also, its fluorescence was widely spread to the brain over time, suggesting the involvement of CSF in its delivery. On the other hand, the nanoparticle with hydrophobic peptide was highly focused on the olfactory bulb in the forebrain, and there was no drug movement to the hindbrain. These results suggest that the hydrophobic peptide increased the adhesiveness with the nasal epithelium and increased the residence time in the olfactory bulb [146]. The study supports the hypothesis that the lipophilicity of the nanoparticles may control the drug delivery pathway.

## 8. Therapeutic Applications of Nose-To-Brain Delivery

### 8.1. Epilepsy

Epilepsy is a chronic neurological disease that causes seizures and can be manifested at all ages, though the highest numbers of new cases occur in childhood and the geriatric population [147]. Epilepsy affects more than 65 million people globally, and about 4.6 million people are diagnosed each year [148,149]. According to the International League Against Epilepsy, there are six etiologies of epilepsy: (1) structural, (2) genetic, (3) infectious, (4) metabolic, (5) immune, and (6) unknown [150]. The cause of epilepsy is not limited to one specific etiology, as they can be combined. Also, the most common causes are different according to population and area. For example, children are more likely to suffer seizures from genetic disorders, whereas from the older generation it can be from an acquired injury. These physiological changes alter the number and properties of voltage or ligand-gated ion channels in the neuronal membrane and lead to hyperexcitation of neurons and, ultimately, a seizure [151]. The symptoms of epilepsy can differ based on the region of the brain and types of seizures. The symptoms include motor symptoms, such as twitching or shaking, sensory symptoms, such as numbness and tingling, and loss of consciousness. If the clinical and/or electrographic seizure lasts more than 5 min, it is called status epilepticus. This serious condition can cause severe morbidity and mortality [152]. Moreover, the elderly population can develop multiple complications such as fractures, depression, and anxiety. The general approach to treat epilepsy is antiseizure medications and benzodiazepines, but these agents are symptomatic treatments only [148]. Typical antiepileptic agents have many drug-drug interactions, as they can modify hepatic enzymes such as CYP3A4 and CYP1A2. This is significantly more problematic in the geriatric population, as they usually take multiple medications to control their chronic disease [153].

Nasal administration of antiepileptics is attractive since it can be administered relatively easily and has good compliance by avoiding parenteral injection. Also, it can decrease drug interactions, hepatic degradation and reduce systemic side effects. One of the challenges to delivering these antiepileptics and benzodiazepines is that they have limited water solubility, which may prevent effective doses to the brain [154]. Many different formulations have been studied to avoid this obstacle, but only nanoparticles were reviewed in this article. Table 1 summarizes various applications of nanoparticles for the treatment of epilepsy.

Samia et al. formulated a nanoemulgel with carbamazepine (CBZ), an anticonvulsant that is one of the most prescribed medications for epilepsy [155]. Nanoemulgel is a combination of nanoemulsion and gel matrix, which acts as a drug reservoir and prevents enzymatic degradation [163]. One of the major drawbacks of CBZ is its side effect profile, which can cause dermal, hematologic, hepatic, pulmonary, renal, and ocular toxicity [164,165]. To increase the drug targeting effect to the brain, the authors used an *o/w* nanoemulgel system that contains oleic acid/labrasol in a ratio of 1:5 and xanthan gum, a mucoadhesive polymer. Xanthan gum is a high molecular weight anionic hydrophilic polymer and acts as a thickener and stabilizer of emulsions or suspensions due to its excellent heat and pH tolerability [166]. The particle size was between 45–146 nm and had high bioadhesion strength (0.142N) to the bovine nasal mucosa. Although it had a slow in vitro release pattern, 65% of carbamazepine was released in 1 h. The onset of convulsion and death time of pentylenetetrazole treated mice was more favorable in the nanoemulgel IN than CBZ IV, but the statistical significance was not given between the groups. Also, CBZ nanoemulgel IN showed similar efficacy to CBZ IV solution in preventing immediate clonic convulsion [155]. This study showed that CBZ nanoemulgel IN could be a promising brain targeting delivery system for the treatment of epilepsy.

Sharma et al. designed polymer-based NPs that encapsulate diazepam (Dzp). Dzp is a fast onset and long-lasting benzodiazepine widely used as an anxiolytic, hypnotic, and antiepileptic [167]. Due to its lipophilicity, Dzp is easily redistributed out of the brain, so the serum level of Dzp is decreased in the brain. To maintain therapeutic efficacy, multiple dosing of Dzp is required. To provide controlled release of Dzp and minimize systemic side effects from multiple dosing, this study adopted the N2B delivery system and used PLGA as a carrier. The authors first optimized the formulation using Box-Behnken design with PLGA, poloxamer 407, *w/o* phase ratio, drug concentration as independent variables to evaluate z-average, drug EE%. By using different ratios, the study used 32 mg/mL PLGA, 15 mg/mL poloxamer, 6:1 *w/o* phase ratio, 5 mg/mL drug concentration to have 183.2 nm and 87.8% drug EE. Ex vivo drug release study showed 18.2 ± 2.2% initial release in 2 h, and 64.4 ± 1.8% sustained release in 24 h. Also, PLGA-Dzp showed higher cell viability compared to Dzp solution (79 ± 1.2% vs. 74 ± 1%), though the statistical significance was not shown. Biodistribution studies of technetium-99m-labeled (99mTc) Dzp showed that PLGA-Dzp IN had a higher brain/blood concentration ratio of the drug than Dzp solution IV and IN up to eight hours post-administration (*p* < 0.05). The PLGA-Dzp IN had significantly higher DTE and DTP than Dzp IN in mice (258% vs. 125% and 61.3% vs. 1% respectively) [156]. This study suggests that PLGA-Dzp could potentially be used for the treatment of epilepsy.

Similarly, PLGA NP loaded with oxcarbazepine was evaluated for N2B delivery [157]. Oxcarbazepine (OX) is a structural analog of carbamazepine and has a better pharmacokinetic profile and less drug-drug interaction than carbamazepine. Nonetheless, it still has side effects due to its high distribution profile which causes reduced sodium and bone density [168]. PLGA OX NP had a size of 256.16 ± 2.94 nm, ZP of -15.12 ± 0.36 mV, PDI of 0.144 ± 0.024, and EE of 85.1 ± 2.1%. After two years of storage as freeze-dried powder, the formulation was stable, with a slightly increased size from 256.16 nm to 294.2 nm. Fluorescent PLGA NPs showed that 5% of the instilled dose was detected in the brain 3 h after the intranasal administration, and more than 8% of the drug was measured in the brain 24 h after the second IN instillation. The drug concentration was only detected in the CSF but not in the bloodstream 3 h post-intranasal administration, which confirms the nasal absorption of PLGA nanoparticles. A daily dose of PLGA-OX IN was given for 11 days as a pre-treatment, and it significantly reduced the occurrence and the duration of symptoms based on the Racine’s Convulsion Scale (RCS) in pentylenetetrazole-induced rats. The expression of neural markers, neurofilament, and beta-tubulin was increased, whereas expression of caspase activity was decreased in the rats pretreated with PLGA-OX compared to those of untreated rats [157].

Using the same drug, El-Zaafarany et al. formulated Tween 80-coated emulsomes using triolein for triglycerides [158]. An emulsome is similar to a liposome, but the main difference is that a liposome has an aqueous inner core, whereas an emulsome has a solid lipid inner matrix, which allows for the loading of hydrophobic molecules in lipid bilayers and a solid inner core [169]. The authors first optimized the formulation using different ratios of phosphatidylcholine (PC) and triglycerides (TG) and different types of lipids (triolein, tripalmitin, tristearin, compritol). Then, based on size, ZP, EE, and drug release, Tween 80 coated emulsome with PC: TG (3:1) ratio and triolein were selected. This emulsome showed no histopathological alteration in rat nasal epithelium but caused mild inflammation in the nostrils. The OX emulsome IN had higher systemic absorption due to its lipophilic nature, and significantly higher brain AUC and shorter Tmax than OX PO and OX IV. DTE of OX emulsome IN 265.7%) and DTP of OX emulsome IN (62.3%) suggest the efficiency of direct brain targeting through the olfactory pathway, rather than indirect systemic pathway from intranasal administration [158]. OX can be a drug candidate for N2B delivery, since several carriers have successfully delivered it to the brain.

### 8.2. Alzheimer’s Disease

Alzheimer’s Disease (AD) is a chronic neurological disease and accounts for 60–80% of diagnosed dementia [170]. In the United States, about 5.8 million people were diagnosed with AD in 2020, accounting for 10% of the geriatric population [171]. By 2050, it is estimated that 1 in 5 people will be older than 65, and the number of AD patients will be 13.8 million [172]. Although AD is the fifth leading cause of death in the elderly, dementia does not directly cause mortality. Rather, the most common cause of death in AD patients is pneumonia and ischemic heart disease [173].

The exact etiology of AD is still unknown, but some genetic and environmental factors have been identified. Mutations of three genes, presenilin 1, 2, and amyloid precursor proteins, are responsible for early-onset familial AD. Apolipoprotein E, on the other hand, is responsible for late-onset AD [174]. These proteins are responsible for beta-amyloid plaques aggregation and the hyperphosphorylation of tau proteins, which are believed to be the two most significant signs of AD [175].

The symptoms of AD can be either cognitive (memory loss, aphasia) or behavioral (depression, physical or verbal aggression). The former tends to be present throughout the stages, whereas the latter is less predictable [176]. For the treatment of AD, cholinesterase inhibitors and N-Methyl-D-aspartate (NMDA) receptor antagonists are used, but they are all symptomatic relief and do not prevent neurodegeneration nor cure the disease [177]. Various strategies have been incorporated to treat AD, including different classes of medications that are not clinically approved for the treatment of AD, novel carriers, and different dosage forms. Table 2 summarizes the studies using N2B delivery methods for the treatment of AD.

Many studies focused on cholinesterase inhibitors to efficiently target the brain through the intranasal pathway. Bhavna et al. used donepezil-loaded chitosan nanosuspension to evaluate in vitro and in vivo safety of the N2B delivery system [178]. Donepezil is a cholinesterase inhibitor, and it is a commonly used medication to treat mild, moderate, and severe AD. The particle size is between 150–200 nm and it has an EE in the range of 92–96%, with drug loading capacity in 40–48%. No significant differences were found between 0.5 mg/mL, 1 mg/mL, and 1.5 mg/mL of donepezil nanosuspension in terms of body weight, hematological values, and histopathology in rats. Also, no toxicity was found in the nasal mucosa and brain with the treated mice. AUC_0_–_24 h_ (brain) of donepezil nanosuspension IN was higher than that of free donepezil IN (440.20 ± 10.64 vs. 95.216 ± 8.52 ng/mL). This study showed an effective and safe N2B delivery of donepezil-loaded nanosuspension.

Sunena et al. used another class of cholinesterase inhibitor, galantamine, loaded into nanoparticles with thiolated chitosan (TC) to increase mucoadhesion and bioavailability in the brain. The nanoparticle was prepared by electrostatic crosslinking of TC with tripolyphosphate pentasodium (TPP). A pharmacodynamic study was performed by evaluating the reversal of scopolamine-induced amnesia in mice. Galantamine-TC NP IN significantly decreased transfer latency in the Elevated Plus Maze compared to both the scopolamine group and scopolamine with blank NPs treated group. Also, galantamine-TC NP IN significantly decreased the time spent in the target quadrant in Morris Water Maze models and decreased acetylcholine esterase activity compared to galantamine IN solution. N2B delivery of galantamine-loaded TC NP has a promising result by increasing memory function and preventing acetylcholinesterase activity [180].

Using the same drug, Li et al. formulated liposomes with propylene glycol (PG) for N2B delivery. The average size of the liposomes was 112 ± 8 nm, the ZP was −49.2 ± 0.7 mV, and the EE was 83.6 ± 1.8%. An in vivo study in rat brains showed that galantamine liposome IN significantly decreased the acetylcholinesterase activity and increased galantamine concentration in the brain when compared with galantamine PO and IN solution (*p* < 0.05) [181].

Several studies showed promising results with non-approved medications for the treatment of Alzheimer’s disease. Jojo et al. used NLC to encapsulate pioglitazone (PIO), a peroxisome proliferator-activated receptor-gamma agonist. It is commonly prescribed for the treatment of type II diabetes, but recently many studies have shown the potential effect in treating Alzheimer’s disease by reducing oxidative stress [184,190,191,192]. The benefit of encapsulating PIO is that it can limit systemic side effects such as peripheral edema and bladder cancer and bypass the BBB to get absorbed [193]. The study used capmul MCM as a liquid lipid, tripalmitin as a solid lipid, and PF68 and Tween 80 as surfactant. The particles had a size of 211.4 ± 3.54 nm, ZP of 14.9 ± 1.09 mV, PDI of 0.257 ± 0.108, and EE of 70.18 ± 4.5%. An in vitro drug release study showed biphasic release, an initial fast release followed by sustained release. A cytotoxicity study with immunohistochemistry showed very little toxicity on the nasal epithelium of sheep nasal mucosa and neuronal cells. Another in vivo biodistribution study manifested that the brain/plasma ratio of PIO concentration in PIO NLC IN was significantly higher than that in PIO IN and IV group (1.6 vs. 0.84 vs. 0.15 respectively), which implies reduced potential systemic toxicity [184]. Overall, the study showed the possibility of delivering PIO to the brain, but a detailed analysis of the effectiveness of PIO IN for the treatment of Alzheimer’s disease is still necessary.

**Figure 8:**
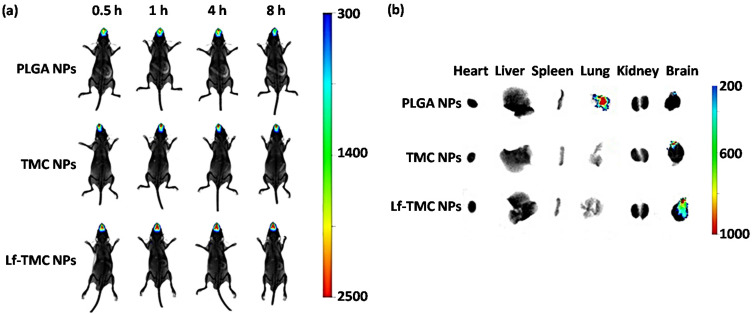
In vivo and ex vivo fluorescence images of organs of mice. (**a**) In vivo imaging of mice at 0.5, 1, 4, and 8 h after treatment with DiR-loaded PLGA NPs, TMC NPs, and Lf-TMC NPs at a dose of 0.5 mg DiR/kg of body weight via the intranasal route. (**b**) Ex vivo imaging of organs excised from mice at 8 h after the intranasal administration. Modified from [185].

Meng et al. used lactoferrin (Lf) and N-trimethyl chitosan (TMC) as ligands to increase the efficiency of Huperzine A-loaded PLGA nanoparticles for the treatment of AD [185]. Huperzine A is a cholinesterase inhibitor that is not approved to be used in AD but can be used as a dietary supplement for memory enhancement. One of the major side effects of Huperzine A is gastrointestinal-related side effects such as nausea, vomiting, constipation, and diarrhea [194]. To decrease the drug’s systemic side effects and achieve better brain targeting, the study used the N2B delivery method. The nanoparticles had a particle size of 153.2 ± 13.7 nm, ZP of +35.6 ± 5.2 mV, PDI of 0.229 ± 0.078, and EE of 73.8 ± 5.7%. Absorption of mucin to Lf-TMC nanoparticle was 86.9 ± 1.8%. This NP showed sustained release over 48 h and higher cellular uptake of Lf-TMC nanoparticles. Also, an in vivo study of Lf-TMC Huperzine A nanoparticles showed higher fluorescence intensity, longer residence time, and higher specificity to the brain than TMC nanoparticles and PLGA nanoparticles (Figure 8). Interestingly, the study further analyzed the drug distribution to the specific areas of the brain (olfactory bulb, cerebrum, cerebellum, hippocampus) and showed that AUCs of Lf-TMC NPs IN in all areas were significantly higher than those of TMC NPs IN and PLGA NPs IN [185]. The presence of Lf and TMC substantially increased the mucoadhesion and accumulation of the drug in the brain. Therefore, such nanoparticles can potentially be used for the effective N2B delivery.

### 8.3. Parkinson’s Disease

Parkinson’s disease (PD) is a progressive neurodegenerative disorder associated with motional and cognitive function. It is the second-highest prevalent neurodegenerative disease after Alzheimer’s disease [195]. As the count of countries affected by an aging society has been growing, the number of people diagnosed with PD is also growing. The global prevalence and number of deaths associated with PD more than doubled from 1990 to 2016 [5]. Some of the risk factors of PD include aging, genetic polymorphism, and environmental factors such as increased exposure to pesticides [196]. The exact etiology of PD is still unknown, but the loss of dopaminergic neurons from substantia nigra pars compacta to the striatum is one of the key characteristics of PD. A total 30% loss of dopamine neurons in substantia nigra can manifest symptoms of PD [197]. Another hallmark of PD is Lewy bodies, which are abnormal protein aggregates that can lead to neuronal degeneration [198]. PD is manifested by the presence of bradykinesia and at least one of the following: resting tremor, rigidity, or postural instability. The general approach of PD treatments includes dopamine precursor and dopamine agonists for dopamine repletion, monoamine oxidase B (MAO-B) inhibitors for preventing dopamine breakdown in the brain, and anticholinergics for tremor management. These treatments are for symptomatic relief, not for the cure of the disease, and still entail many systemic side effects and lack of efficacy [196,199]. To improve current pharmacologic therapy, many different dosage forms have been studied, and N2B delivery is one of them (Table 3).

Mittal et al. used chitosan glutamate nanoparticles (CGNPs) to encapsulate rasagiline (RAS), a selective irreversible second-generation MAO-B inhibitor with dopamine receptor agonist activity [200]. RAS oral formulation can induce GI side effects such as nausea, vomiting and has a short half-life with low oral bioavailability due to hepatic first-pass metabolism [214]. Also, CGNP can increase the dissolution rate of the drug by decreasing drug crystallinity and increasing the drug’s solubilizing effect [215]. The study first underwent optimization using the Box-Behnken design, and 0.15% of chitosan glutamate, 0.2% of sodium triphosphate, and 0.15% of rasagiline were chosen to yield the size of 151.1 ± 10.31 nm, PDI of 0.380 ± 0.01, and EE of 96.43 ± 4.23%. In vitro release study showed initial rapid release of RAS in 1 h with sustained and controlled release throughout 24 h. An ex vivo permeation study using goat nasal mucosa showed a higher cumulative amount of drug permeated and percentage of drug permeated through nasal mucosa in RAS CGNPs group when compared with RAS solution (12.5 ± 0.053 μg/cm^2^ vs. 4.69 ± 0.059 μg/cm^2^ and 62.25 ± 0.41% vs. 23.45 ± 0.38% respectively). An in vivo pharmacokinetic study showed that maximum concentration was achieved in RAS CGNPs IN group in 15 min whereas (992.25 ± 31.17ng/mL) RAS solution IN and RAS CGNP IV in 30 min (634.23 ± 34.93 ng/mL and 346.74 ± 19.68 ng/mL respectively). DTE and DTP of RAS CGNPs showed in average 325 ± 40% and 69.27 ± 2.1%, which represents better efficient brain targeting through IN than IV [200]. It showed that CGNP could be a promising carrier to deliver rasagiline to the brain, although it requires more data to evaluate its efficacy.

Tang et al. formulated borneol and lactoferrin co-modified nanoparticles (Lf-BNPs) with dopamine to enhance permeability and specificity to the striatum [201]. Borneol is a bicyclic monoterpene and can increase penetration of other drugs in the nasal mucosa and the blood-brain barrier [216]. The lactoferrin receptor is highly expressed in the apical surface of respiratory epithelial cells, capillaries, and neurons of the neurodegenerative brain [185]. The particle size was 175.3 ± 9.6 nm, ZP of −15.7 ± 0.86 mV, PDI of 0.129 ± 0.011, and EE of 25.43 ± 5.32%. Lf-BNPs with dopamine did not decrease the cell viability of SH-SY5Y and 16BHE cells, but free dopamine with the same concentration significantly decreased it. Cellular uptake of Lf-BNPs was highest compared to Lf-NPs and dopamine NPs (*p* < 0.05). A pharmacokinetic study showed that AUC_0-12h_ in the brain was significantly higher in dopamine-Lf-BNPs than Lf-NPs and dopamine NPs. Although the presence of borneol did not significantly affect the maximum dopamine concentration, it decreased the time to reach maximum dopamine concentration in the brain (15 min vs. 1 h). Evaluation of contralateral rotation behavior in apomorphine-induced rats showed that Lf-BNPs decreased rotations significantly compared to control and dopamine NPs. Also, the content of dopamine and its metabolites in the striatum was highest among the groups, which indicates that dopamine levels could be effectively restored [201]. This study showed the great potential of using borneol to increase penetration of nasal mucosa and using lactoferrin to increase the delivery efficacy.

Md et al. formulated bromocriptine-loaded chitosan (CS) nanoparticles for N2B delivery to enhance the efficiency of drug delivery to the brain [202]. Bromocriptine (BRC) is an ergot-derivative dopamine receptor agonist used as an adjunctive treatment to levodopa. The main downfall of this drug is the rapid hydroxylation in the liver, which leads to low bioavailability in the brain [217]. To overcome this obstacle, BRC was encapsulated in chitosan nanoparticles and administered intranasally. The BRC-CS NPs had a particle size of 161.3 ± 4.7 nm, ZP of +40.32 ± 2.78 mV, PDI of 0.44 ± 0.03, EE of 84.2 ± 3.5%, and the ratio of chitosan to TPP was 3.3 to 1. A biodistribution study of BRC-CS NPs IN showed a higher accumulation of BRC in the brain when compared with BRC solution IN and BRC-CS NPs IV. Also, BRC-CS NPS IN had a considerable accumulation of BRC in the stomach and intestine, whereas BRC-CS NP IV was distributed among different organs, including liver, lung, and spleen. The efficacy study was evaluated by haloperidol-induced catalepsy and akinesia in mice using five different groups: saline treated mice (G1), mice treated with free non-bound haloperidol (G2), BRC solution PO (G3), BRC solution IN (G4), BRC-CS NPs IN (G5). G3 and G4 groups decreased the catalepsy and akinesia events compared to G2, but BRC-CS NPs significantly decreased these events to the extent that catalepsy and akinesia were not statistically significant compared to the control group (G1). The relative bioavailability of BRC-CS NP IN compared to IV was 135.7 ± 14.05%, demonstrating that higher BRC could be reached when administered IN (*p* < 0.05). Also, DTE and DTP values of BRC-CS NP IN (633 ± 86.1% and 84.2 ± 1.9%, respectively) showed the preference of IN over IV [202]. Biodistribution and efficacy studies demonstrated that BRC-CS NP IN could potentially be an effective non-invasive option for treating Parkinson’s disease.

**Figure 9:**
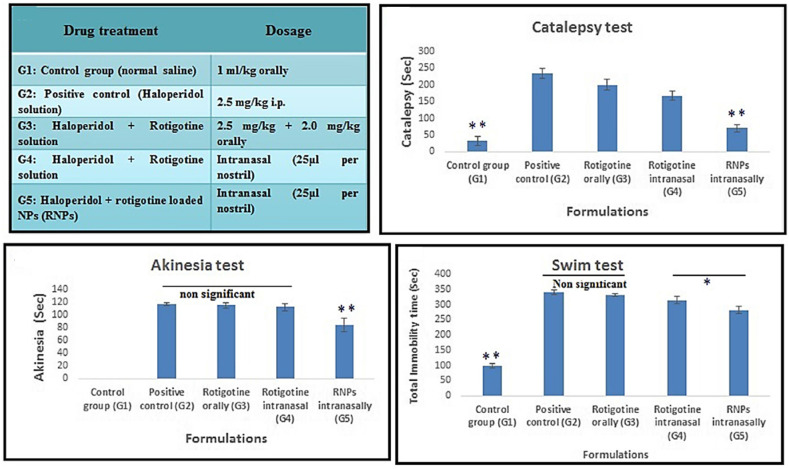
Effects of rotigotine and RNPs on rats with Parkinson’s disease induced by haloperidol. The data were analyzed using one-way ANOVA followed by Tukey’s post hoc test. *: *p* < 0.05 and ** *p* < 0.01 compared to positive control. Means ± SD (*n* = 4) are shown. Reproduced with permission from [203], Elsevier, 2020.

Bhattamisra et al. developed chitosan NPs loaded with rotigotine, a non-ergot dopamine agonist, and evaluated its antioxidant and neuroprotective activities [203]. Rotigotine has very poor bioavailability when taken PO due to extensive hepatic metabolism, so only transdermal administration is used clinically [218]. Rotigotine-loaded chitosan nanoparticles (RNPs) had a particle size of 75.37 ± 3.37 nm, ZP of +25.53 ± 0.45 mV, PDI of 0.368 ± 0.02, and EE of 96.08 ± 0.01%. A fluorescent study of coumarin-6-NP showed rapid cellular internalization and was well distributed in the cytoplasm. RNPs showed significant neuroprotective activity in 6-hydroxydopamine (6-OHDA) induced cells by increasing the viability, whereas placebo nanoparticle or rotigotine solution did not. Also, RNPs significantly decreased the expression of α-synuclein and reactive oxygen species and increased the expression of tyrosine hydroxylase and superoxide dismutase in 6-OHDA induced cells compared to the positive control. Since increased α-synuclein activity is a major feature of Parkinson’s disease etiology, RNPs demonstrated some neuroprotective activities [219]. An akinesia test, catalepsy test, and swim test were carried out on haloperidol-induced catalepsy in rats to evaluate RNPs efficacy. RNPs IN significantly decreased the duration of catalepsy, akinesia, and total immobility time during the swim test (Figure 9). In addition, the treatment significantly decreased lactate dehydrogenase levels and increased catalase activity (*p* < 0.05). A pharmacokinetic study showed that RNPs (IN) increased the rotigotine maximum concentration in the brain when compared with rotigotine solution IN, RNPs IV and rotigotine PO (61.72 ± 7.44 ng/mL vs. 36.74 ± 23.41 ng/mL; 36.92 ± 13.87 ng/mL and 5.35 ± 0.39 ng/mL, respectively). Higher DTE and DTP were obtained from RNPs IN (258.10 ± 17.13% and 53.87 ± 10.14%) than rotigotine solution IN (187.9 ± 13.33% and 46.62 ± 3.80%) [203]. Data obtained in behavioral and biochemical studies support the potential use of rotigotine for N2B delivery. To further evaluate its clinical efficacy and safety, a direct comparison with transdermal rotigotine would be necessary.

### 8.4. Stroke

Stroke is the second leading cause of death and the third leading cause of chronic disability worldwide [220]. Of all strokes, 87% are ischemic and 13% are hemorrhagic stroke [221]. Ischemic stroke arises either due to local thrombus or emboli, leading to the blockage of a cerebral artery. This arterial occlusion results in decreased cerebral blood flow and requires intervention within 2 to 3 h [222]. The only clinically available intervention is IV tissue plasminogen activator (tPA), which helps break down the clots and help restore blood flow to the brain [223]. On the other hand, hemorrhagic stroke is caused by the rupture of the cerebral vessels that increases pressure on adjacent cells. Although many different medications such as antihypertensive drugs, osmotic agents, and anticoagulants are used to manage hemorrhagic stroke, there is no single medication approved to treat hemorrhagic stroke [224]. Due to limited options to treat both types of strokes, N2B delivery has been studied among other different approaches for managing stroke (Table 4).

Xiao et al. evaluated the efficacy of thymoquinone-loaded PLGA chitosan nanoparticles for cerebral ischemia in mice [225]. Thymoquinone (TQ) is a phytochemical extracted from seeds of *Nigella sativa* that can decrease damage triggered by reactive oxygen species [228]. However, TQ is light sensitive and hydrophobic, with poor solubility in an aqueous solution, causing its poor bioavailability. TQ-loaded PLGA-NPs were prepared using the emulsion solvent evaporation technique. The size of NP was 183.5 ± 8.2 nm, PDI of 0.257 ± 0.02, ZP 33.63 ± 2.25, EE of 73.2 ± 2.6%, and loading capacity was 31.4 ± 2.1%. It showed a biphasic release pattern with 16% release in 1 h followed by 88.21 ± 2.872% sustained release over 24 h. The middle cerebral artery occlusion (MCAO) model was chosen to evaluate the efficacy of TQ-PLGA NPs. Although the evaluation between the treatment groups was not performed, the locomotor activity of TQ PLGA NPs IN treated rats was significantly improved compared to MCAO control rats (*p* < 0.01). Also, the catalase activity and superoxide dismutase activity were significantly increased in TQ PLGA NP IN group when compared with the control. A pharmacokinetic study in the brain and plasma showed that TQ-PLGA NPs IN achieved higher AUC, Cmax, and longer half-life than TQ-PLGA NPs IV. Based on the improved DTE and DTP one can conclude that TQ PLGA NPs could be directly delivered to the brain via IN (524.17% and 80.47%, respectively) [225].

**Figure 10:**
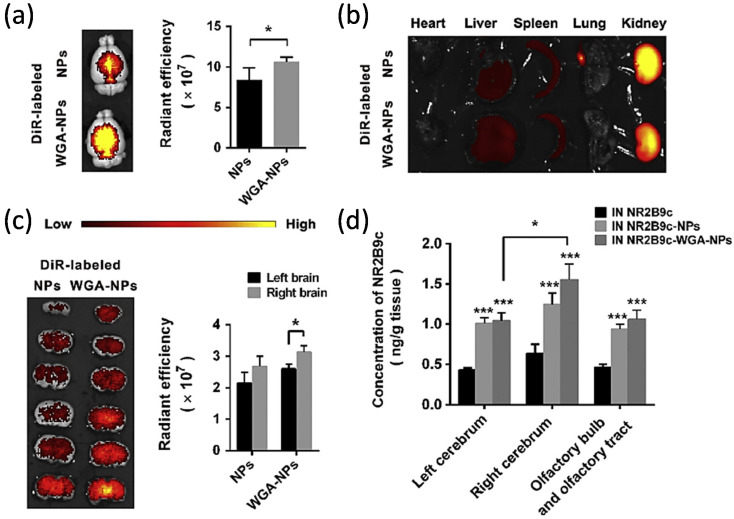
Biodistribution of WGA-NPs and NR2B9c loaded into WGA-NPs IN in the rats with stroke model. (**a**) Biodistribution of DiR-labeled NPs and WGA-NPs in brain. Ex vivo fluorescence brain images (**left**) and bar graft (**right**, *n* = 4). (**b**) Ex vivo fluorescence imaging of peripheral organs. (**c**) Fluorescent images of brain slices (**left**) and bar graph (**right**, *n* = 4) (**d**) Biodistribution of NR2B9c at 1 h after intranasal administration with NR2B9c, NR2B9c NP or NR2B9c-WGA NP in different brain areas of rats. A, C and D: * *p* < 0.05, between groups; D: *** *p* < 0.001, when compared with NR2B9c IN in the same brain area. Reproduced with permission from [226], Elsevier, 2019.

Li et al. used NR2B9c-loaded wheat germ agglutinin (WGA) PEG-PLGA nanoparticles to evaluate neuroprotective effect for the treatment of stroke [226]. NR2B9c is a peptide that prevents the interaction between N-Methyl-D-aspartate receptors (NMDARs) and the postsynaptic density protein-95, inhibiting neurotoxic signaling pathways. Also, it helps to decrease infarction volume and ischemic brain damage up to 3 h after the stroke onset [229]. Although NR2B9c possesses many promising properties for the treatment of stroke, its hydrophilicity and a molecular weight of 977 Da limit the drug penetration through the cell membrane and BBB after the PO or IV administration. To increase N2B delivery and target nasal epithelium, a non-immunogenic and nontoxic WGA glycoprotein that binds to N-acetyl-D-glucosamine and sialic acid residues in the nasal epithelium was used [230]. With PEG and WGA incorporation, the NPs were expected to have increased mucus penetration and a better brain targeting effect. The NPs had a mean size of ~139 nm, PDI < 0.2, ZP of −23.3 mV, EE of ~50%, drug loading of ~1.5%, and WGA conjugation efficiency of 59.99 ± 2.61%. NR2B9c remaining in plasma was in most cases significantly higher in NR2B9c-WGA NP than in NR2B9c NP and free NR2B9c. In nasal wash fluid, however, a difference in the concentration of NR2B9c was not statistically significant between WGA-modified and unmodified nanoparticles but significantly higher (*p* < 0.001) when compared with free NR2B9c. Cytotoxicity studies of nanoparticles in Calu-3 cells and primary cortical neurons showed no major damage up to 1 mg/mL concentration. Also, TNF-α levels in rat olfactory bulbs, brain, and peripheral organs did not significantly change. Cellular uptake of fluorophore 5-carboxytetramethylrhodamine (5-TAMRA) conjugated to NR2B9c showed that NR2B9c-WGA NP had significantly higher cellular uptake than free NR2B9c and NR2B9c NP (*p* < 0.001 and *p* < 0.05, respectively). Also, the neuroprotective effect of NR2B9c-WGA NP was demonstrated by inhibition of NMDA-induced LDH leakage and nuclear chromatin condensation in primary cortical neurons. These in vitro results supported the pharmacokinetic study and showed that NR2B9c-WGA NP IN and NR2B9c NP IN delivered more NR2B9c to the brain tissue than free NR2B9c IN. The peak concentration of NR2B9c was achieved in 30 min in the olfactory bulb or olfactory tract and 1 h in different parts of the brain such as the cerebrum, hippocampus, and cerebellum. DTE of NR2B9c-WGA NP in the cerebrum and olfactory bulb were above 150% as well as DTP in the cerebrum (78.58%) and olfactory bulb (71.90%) make these nanoparticles suitable for the brain delivery via N2B pathway. A similar distribution pattern was observed in rats with middle cerebral artery occlusion (MCAO), where NR2B9c-WGA NP delivered a significantly greater concentration of NR2B9c than free non-bound NR2B9c. Interestingly, more significant nanoparticles accumulation was observed in the occluded right hemisphere than the undamaged left hemisphere, indicating delivery of NR2B9c to the damaged brain (Figure 10). Also, NR2B9c-WGA-NP significantly decreased infarcted area and neurological scores compared to other formulations in MCAO model rats, which showed its neuroprotective effects in vivo [226]. Consequently, intranasal administration NR2B9c-WGA NP potentially can be an efficient and safe method for the treatment of ischemic stroke.

### 8.5. Schizophrenia

Schizophrenia is a chronic psychiatric disorder that is manifested by many different symptoms such as delusion, hallucination, and abnormal behavior. The prevalence of schizophrenia is 1% internationally and typically occurs during adolescence [231]. Its etiology is still unknown, but it is considered to be caused by multiple environmental and genetic factors that disrupt brain function. It is hypothesized that genes controlling N-methyl-D-aspartate (NMDA) receptor activity dysfunction, neurotransmitter dysfunction, infection, and inflammation may play a role in the development of schizophrenia [232]. Pharmacotherapy is the mainstream treatment of schizophrenia along with a psychosocial rehabilitation program. First-generation antipsychotics (FGAs), also called typical antipsychotics, are dopamine receptor antagonists, and second-generation antipsychotics (SGAs) or atypical antipsychotics are serotonin-dopamine antagonists [233]. SGAs have a lower risk of neurologic side effects than FGAs, but some SGAs have metabolic and cardiovascular side effects, so the pharmacotherapy should be optimized individually based on patient medical history [234]. To decrease these systemic risks and increase bioavailability of antipsychotic drugs for the brain, several different approaches have been adopted, and intranasal administration is one of them [235,236] (Table 5).

Shah et al. developed quetiapine-loaded chitosan nanoparticles and optimized them using the Box-Behnken design [237]. Quetiapine fumarate (QF) is an SGA and a combination of dopamine type 2 (D2) and serotonin type 2 (5-HT2) antagonist, which causes fewer extrapyramidal symptoms [244]. The limitations of this drug include a short half-life and poor oral bioavailability due to the first-pass liver metabolism [245]. Also, quetiapine is a substrate for P-glycoprotein (P-gp) drug efflux pumps, leading to decrease of its concentration in brain cells [246]. The authors used chitosan nanoparticles to overcome these obstacles and evaluated the efficacy and safety of its intranasal administration. The nanoparticle was made using the ionic gelation method, and optimized NP had a size of 131.08 ± 7.45 nm, PDI of 0.252 ± 0.064, ZP of 34.4 ± 1.87 mV, and EE of 89.93 ± 3.85%. Nasal diffusion study with goat mucosa showed that QF-NP led to 60% diffusion after 6 h of administration, whereas free QF solution caused 35% diffusion. Nasal histopathology revealed no significant structural damage or cell death induced by QF-NP. In a pharmacokinetic study, the concentration of QF in the brain was highest in QF-NP IN followed by QF-NP IV and free QF IN, which indicates that IN administration of QF in NP enhanced QF delivery to the brain. Both QF-NP IN and free QF IN showed lower plasma concentration at all times than QF-NP IV, which indicates lower systemic circulation and possible less systemic side effects with IN administration. DTE and DTP of QF-NP were 374.93 ± 15.02% and 73.33 ± 4.14%, respectively, which depicts higher efficiency of intranasal brain delivery of QF [237]. Overall, QF-NP showed a promising result of intranasal delivery of QF, though it requires efficacy data to be potentially used for the treatment of schizophrenia.

Similarly, another atypical antipsychotic was evaluated for N2B delivery. Sawant et al. used aripiprazole-loaded polycaprolactone nanoparticles for intranasal delivery [239]. Aripiprazole (APZ) is a third-generation atypical antipsychotic with a high affinity to D2 and 5-HT2 receptors. Due to its extensive hepatic metabolism and being the P-gp substrate, APZ requires dose-escalation to maintain its treatment effect which in turn can induce many systemic side effects such as QTc prolongation, hyperglycemia, and hypotension [247]. Polycaprolactone was used for nanoparticle formulation due to its high stability and cellular uptake with low toxicity [248]. The optimized nanoparticle had a size of 199.2 ± 5.65 nm, ZP of −21.4 ± 4.6 mV, EE of 69.2 ± 2.34%. An in vitro drug release study showed sustained release of APZ (45.60 ± 3.15%) in 8 h and overall release of 89.51 ± 3.11% after 72 h. A histopathological study demonstrated that free APZ solution caused some cilia damage, whereas APZ-NP did not significantly damage the nasal epithelium. An in vivo pharmacokinetic study demonstrated that APZ-NP IN led to higher Cmax and AUC in the brain than APZ-NP IV [239]. However, although, in general, intranasal administration of APZ-NP increased the brain targeting effect compared to IV administration, the DTE value (64.11 ± 4.68%) did not support the author’s conclusion.

Fonseca et al. also used caprolactone nanoparticles to encapsulate an atypical antipsychotic, olanzapine (OLA). On the surface of the nanoparticles, amphiphilic methacrylic copolymers (methyl methacrylate and 2-(dimethylamino)ethyl methacrylate) were functionalized to make the particles surfactant-free and mucoadhesive. The characterizations of NP include the size of 254.9 ± 12.1 nm, zeta-potential of +22.2 ± 1.2 mV, PDI of 0.03 ± 0.01, and EE of 99.00 ± 0.05% at pH 7.4. The increased mucoadhesive property of methacrylic copolymers was confirmed with a higher force required to detach the mucosa after 30 s of contact when compared with unmodified NPs. The OLA-NP had improved by 40% nasal retention compared to free olanzapine. In vivo study in rats showed that OLA-NP increased OLA concentration in the brain 1.55 times higher than free OLA solution (*p* < 0.05). Also, OLA-NP successfully prevented pre-pulse inhibition deficits in apomorphine-induced rats, whereas free olanzapine and blank nanoparticles did not. Pre-pulse inhibition can be a biomarker of schizophrenia as schizophrenia patients have impaired pre-pulse inhibition [249]. No significant nasal toxicity was found after daily administration of OLA-NP (7 days), which suggests its potential safety [240]. The amphiphilic methacrylic copolymer can be a safe and promising carrier to increase mucoadhesive property for drugs with low brain bioavailability.

### 8.6. Depression

Depression is one of the most prevalent mental diseases affecting more than 250 million people globally, and this number is increasing [250]. The cause of depression can be manifested with many different factors such as defects in neurotransmitters, genetics, or other social and economic factors [251]. The general approach to the treatment of depression is pharmacologic therapy along with non-pharmacologic treatment. Common classes of antidepressants include selective serotonin reuptake inhibitors (SSRIs), serotonin-norepinephrine reuptake inhibitors (SNRIs), and monoamine oxidase inhibitors. These medications help to restore neurotransmitter balance in the brain and decrease depressive symptoms [252]. Although there have been advances in the treatment of depression, 30–40% of patients do not respond to the first-line therapy and suffer from social and occupational difficulties, suicidal thoughts, and hospitalizations [253]. One of the major reasons these antidepressants fail is the presence of the BBB and the expression of drug efflux pumps in brain capillaries, endothelial cells, luminal membranes and caveolae [254]. Since many antidepressants are substrates of these transporters, antidepressant bioavailability in the brain is limited and eventually leads to decrease in clinical efficacy [255]. In order to effectively deliver antidepressants to the brain and enhance their specific activity, many researchers studied N2B delivery of antidepressants to bypass the BBB (Table 6).

Haque et al. investigated the utility of intranasally administered venlafaxine-loaded chitosan nanoparticles to enhance brain delivery compared to intravenous infusion [256]. Venlafaxine is an SNRI antidepressant and is widely used in the clinical setting for the treatment of depression. However, it has a short half-life with a slow onset of action and numerous systemic side effects such as QTc prolongation, hypertension, decreased sexual function, and weight loss, making venlafaxine an agent that can benefit from N2B delivery [260]. Venlafaxine nanoparticles were formulated with the ionic gelation technique using chitosan and TPP. The particle size was 167 ± 6.5 nm, ZP of +23.83 ±1.76 mV, PDI of 0.367 ± 0.045, and EE of 79.3 ± 2.6%. An ex vivo permeation study using porcine nasal mucosal membrane showed that venlafaxine chitosan nanoparticles were able to permeate the membrane three times higher than the venlafaxine solution. The locomotor activity of depressed rats was measured to evaluate antidepressant activity using different routes of administration. Chitosan venlafaxine nanoparticles significantly increased the total swimming and climbing time but decreased the immobility time compared to the control, venlafaxine PO, and solution IN group. DTE (508.59%) and DTP (80.34%) of venlafaxine chitosan nanoparticles show increased mucoadhesiveness, effective delivery of the drug to the brain and together with other data confirm better efficacy in treating depression [256].

Tong et al. used a similar drug, desvenlafaxine succinate (DVF), an active metabolite of venlafaxine, and adopted PLGA-chitosan (CS) system to encapsulate the drug. The size of the nanoparticles was 172.5 ± 10.2 nm, ZP of 35.63 ± 8.25 mV, PDI of 0.254 ± 0.02, EE of 76.4 ± 4.2%, and drug loading of 30.8 ± 3.1%. 34% of DVF was released in 1 h and 76.32 ± 3.54 over 24 h from DVF PLGA-CS NPs at pH 6.0. In forced swim test models, treatment of DVF PLGA-CS NPs IN significantly decreased immobility (*p* < 0.01) and increased the swimming time (*p* < 0.01), climbing time (*p* < 0.05), and locomotor counts (*p* < 0.01) compared to the control group. Also, the reserpine reversal test model showed that DVF PLGA-CS NPs IN significantly reduced reserpine induced immobility and weakly reduced ptosis and diarrhea in rats (*p* < 0.05 in 2,4 h). Biochemical studies showed that DVF PLGA-CS NPs IN significantly increased serotonin and noradrenaline level (*p* < 0.01 and *p* < 0.05, respectively) but did not significantly increase dopamine levels compared to the control group. Since DVF is SNRI, the activity and distribution of DVF in the brain were confirmed. However, no comparison test was performed among other treatment groups such as DVF PO and DVF IN. A pharmacokinetic study was performed using DVF PLGA-CS NPs IV, DVF IN, and DVF PLGA-CS NPs IN and showed higher DVF concentration in the brain from DVF PLGA-CS NPs IN than IV administration (954.56 ± 126.63 ng/mL vs. 396.91 ± 64.34 ng/mL). Also, the elimination rate in the brain was significantly lower in the IN group than in the IV group (*p* < 0.05). DTE (544.23%) and DTP (81.62%) showed that DVF PLGA-CS NPs IN has a better brain targeting effect than other formulations [257].

Bari et al. used buspirone, which is clinically used for general anxiety disorder and has a high affinity to 5-HT1 and 5-HT2 receptors [258,261]. Similar to other antidepressants, buspirone undergoes extensive first-pass metabolism, so the absolute bioavailability is only about 4%. Therefore, it requires multiple dosing and eventually exposes the risk of systemic side effects to the patients [262]. To increase its bioavailability and decrease distribution to non-targeted organs, the authors used a thiolated-chitosan system to encapsulate buspirone and increased mucoadhesive properties. Thiolated-chitosan nanoparticle (TC-NP) had a size of 226.7 ± 2.52 nm, PDI of 0.483 ± 0.031, EE of 81.13 ±2.8%, and loading capacity of 49.67 ± 5.5%. TC-NP had biphasic release; about 50% of the drug was released in 2.5 h, and more than 90% was released in 24 h. The ex vivo permeability study using porcine mucin showed that TC-NP had approximately 15% higher binding efficiency than plain chitosan nanoparticles (CS-NP). The elevated plus maze model was used to assess anxiety-related behavior, and TC-NP showed the longest open arm residence time compared to other groups. The Cmax of buspirone from TC-NP IN (797.46 ± 35.76 ng/mL) was higher than that for buspirone IN or IV (417.77 ± 19.24 ng/mL and 384.15 ± 13.42 ng/mL respectively). DTE and DTP were 78.94 ± 15.31% and 95.9 ± 11.31%, respectively. Overall, intranasal administration of TC-NP showed a promising approach for the treatment of generalized anxiety disorder, but the DTE value (78.94 ± 15.31%) did not support the author’s conclusion. [258].

### 8.7. Other CNS/Neurological Disorders

N2B delivery system has been studied in many different CNS/neurological disorders such as neuropathic pain, multiple sclerosis, and Huntington’s disease. Table 7 summarizes the use of N2B delivery in multiple CNS pathological conditions.

Javia et al. investigated tapentadol hydrochloride-loaded chitosan nanoparticles for pain management [263]. Tapentadol (TAP) is a centrally-acting analgesic and acts as a mu-opioid receptor agonist and noradrenaline reuptake inhibitor [270]. One of the biggest problems with its oral route is that it undergoes extensive first-pass metabolism (32% bioavailability), and 97% of the drug is metabolized to inactive metabolite [271]. Also, tapentadol is a hydrophilic compound, so its brain concentration after the systemic administration is limited due to the BBB [270]. In order to increase drug delivery to the brain and lower side effects, the authors used chitosan nanoparticles (CS-NP) to incorporate tapentadol for N2B delivery. CS-NP was prepared by ionotropic gelation method and underwent optimization process to yield size of 201.2 ±1.5 nm, ZP of 49.3 ± 1.2mV, PDI of 0.201 ± 0.01, EE of 63.49 ± 1.61%, and drug loading of 17.25 ± 1.38%. A drug release study showed biphasic release; 25.01 ± 1.12% drug was released in 2 h and 84.04 ± 1.53% after 28 h. No significant damage was found in goat nasal mucosa after treating with TAP CS-NP. A pharmacokinetic study showed that TAP CS-NP IN had higher Cmax, AUC, half-life, and mean residence time than TAP solution IN. DTE of 321 ± 60% and DTP of 68.85 ± 0.49% of TAP CS-NP show a direct pathway from nose to brain, increasing the CNS availability of tapentadol [263].

In a similar context, Nigam et al. used baclofen-loaded PLGA nanoparticles to treat neuropathic pain [264]. Baclofen (Bcf) is a gamma-aminobutyric acid agonist that reduces excitatory neurotransmitters such as aspartate and glutamate and is clinically used for spasticity. Baclofen oral formulation has a short half-life (2–6 h) that requires frequent dosing and leads to systemic side effects such as hypertension and gastrointestinal and nervous system problems [272]. To prevent systemic side effects and increase baclofen concentration in the brain, the authors used PLGA nanoparticles to encapsulate baclofen. The particle size of Bcf-PLGA-NPs was 124.8 nm, ZP of −20.4 mV, PDI of 0.225, EE of 86.45 ± 1.65%. In vitro release study using simulated nasal fluid and simulated CSF showed 40% release in 5 h and 73% in 24 h and 26% release in 5 h, and 98% in 24 h, respectively. Bcf was radiolabeled with 99mTc and its pharmacokinetics was investigated in vivo. It was shown that 99mTc-Bcf-PLGA NPs IN reached maximum distribution in the brain at 3 h and mostly stayed around the brain, whereas 99mTc-Bcf solution IN was spread to other peripheral organs. C_max_ and AUC_0–24 h_ in the brain was the greatest in 99mTc-Bcf-PLGA NPs IN followed by IV, 99mTc-Bcf solution IN, and IV. DTE (183.85%) and DTP (45.92%) show the advantages of N2B delivery of Bcf nanoparticles and signify increased baclofen absorption and distribution.

Gadhave et al. applied N2B delivery of teriflunomide for the treatment of multiple sclerosis [265]. Multiple sclerosis is an inflammatory disease of the CNS that causes chronic demyelination and axonal damage, leading to a permanent disorder [273]. Teriflunomide (TFM) is an immunomodulatory agent with an anti-inflammatory effect that is clinically approved for the treatment of MS, and it inhibits dihydroorotate dehydrogenase, which catalyzes the conversion of dihydroorotate to orotate in the de novo pyrimidine synthesis. One of the biggest concerns of TFM is systematic side effects, as more than 10% of patients in Phase II and III trials experienced hair loss, hepatotoxicity, and nasopharyngitis [274]. In order to prevent these side effects, intranasal delivery of TFM-loaded NLC was studied, optimized, and evaluated for its safety and efficacy. Melt emulsification and ultrasonication methods were used for the preparation of TFM-loaded NLC using compritol 888 AOT (solid lipid), maisine 35-1 (liquid lipid), gelucire 44/14 (stabilizer), and tween 20 (surfactant). The formulation was optimized using Box-Behnken design with drug, lipid ratio (solid: liquid lipid), and surfactant as response variables. In order to achieve the particle size (99.82 ± 1.36 nm), EE (83.39 ± 1.24%), PDI (0.35 ± 0.01), and drug loading (6.68 ± 0.24%), the composition of NLC was finally adjusted to 20 mg of TFM, lipid ratio (240:60 mg), 800 mg of tween 80, mucoadhesive agent (HPMC K4M 30 mg) and poloxamer 407 (1700 mg). In a simulated nasal electrolyte solution, the cumulative TFM release of TFM-mucoadhesive NLC (MNLC) was 96.44 ± 0.73% in 8 h. Ex vivo study using sheep nasal epithelium showed that 83.01 ± 0.69% of TFM was permeated from TFM-MNLC, whereas 65.13 ± 0.86% of TFM was permeated from TFM-NLC in 8 h. The presence of HPMC K4M increased the mucoadhesive property of NLC. No signs of structural deformation or cilia toxicity were found from TFM-NLC and TFM-MNLC. In vivo study was performed using rats induced with cuprizone, a nerve toxin that damages myelin sheaths and causes neurodegeneration. TFM-NLC PO or TFM-MNLC IN was administered to cuprizone-treated rats to evaluate its efficacy in decreasing abnormal behaviors in an elevated plus maze test. TFM-MNLC IN decreased the number of entries in the open zone and showed greater improvement than TFM-NLC PO (*p* < 0.01). TFM-MNLC IN did not induce any significant morbidity and mortality compared to other groups. Although free TFM caused hepatotoxicity and renal toxicity, TMF-MNLC IN did not cause any significant elevation of hepatic transaminase enzymes and kidney biomarkers (*p* < 0.01) [265]. Therefore, TFM-MNLC delivered to the brain via N2B route potentially can be a promising treatment option for multiple sclerosis.

Bhatt et al. developed rosmarinic acid-loaded SLN for the management of Huntington’s disease [266]. Huntington’s disease is a rare autosomal dominant neurodegenerative disease of CNS caused by elongation of CAG codon in the huntingtin gene, which leads to the development of huntingtin protein (Htt). Htt expression in the brain will cause involuntary movement, cognitive and psychiatric disorders [275]. Rosmarinic acid (RA) is derived from the herb *Rosmarinus officinalis* and is a water-soluble antioxidant with various neuroprotective, antinociceptive activities. It has been studied in various nervous system disorders such as Alzheimer’s disease, Parkinson’s disease, and Huntington’s disease [276]. Due to its hydrophilicity, RA concentration in the brain is limited after parenteral injections or oral administration, so the authors used SLN to incorporate RA and administered it IN. RA-loaded SLN were formulated using the hot homogenization method, and the optimized formulation yielded a size of 149.2 ± 18.2 nm, ZP of −38.27 mV, PDI of 0.290 ± 0.021, EE of 61.9 ± 2.2%. The drug release was controlled with ~50% at 7 h and 97.5 ± 2.9% release at 14 h. Behavioral and biochemical assessments were conducted using 3-nitropropionic acid (3-NP) induced rats, and RA IN, RA-SLN IV, and RA-SLN IN were selected as treatment groups. 3-NP significantly reduced body weight, motor coordination, the locomotor activity of rats and increased the time to reach the goal platform (narrow beam test). RA-SLN IN showed the most significant effect in reversing 3-NP effects compared to all treatment groups (*p* < 0.05). In the biochemical study, RA-SLN IN significantly decreased lipid peroxidation, nitrite concentration, and increased catalase and glutathione activities in 3-NP rats (*p* < 0.05 vs. all treatment groups). The C_max_ of RA-SLN in the brain was 0.284 µg/mL, the half-life of 3.17 h, and AUC was 1.505 µg/mL/h [266]. Therefore, intranasal administration of RA-SLN can be a promising method for the management of Huntington’s disease.

Jain et al. adopted N2B delivery of artemether for the management of cerebral malaria [267]. Cerebral malaria is the most severe neurological complication of infection with *Plasmodium falciparum* malaria. Falciparum malaria is one of the leading causes of morbidity and mortality in tropical countries, affecting about 40% of the world’s population, and more than 1 million deaths occur annually due to severe complications of infection [277]. Artemether (ARM) is an anti-malaria medication that is used for chloroquine-resistant *p. falciparum.* Due to its hydrophobicity, the intramuscular injection of ARM may lead to slow and variable absorption and may not be suitable for treating cerebral malaria [278]. In order to increase its efficiency, the authors used the NLC system to incorporate ARM and evaluated its brain targeting efficiency. The optimized formulation of ARM-NLC had a size of 123.4 ± 3.6 nm, ZP of −34.4mV ± 1.2 mV, drug loading of 10.56 ± 0.59%, and EE of 91.2 ± 2.5%. The ARM-NLC did not show significant toxicities in the brain and nasal epithelium. A pharmacokinetic study showed that ARM-NLC IN had a higher drug concentration in the brain than ARM IN and IV from 1 to 6 h (*p* < 0.001). DTE (278.16%) and DTP (64.02%) characterize NLC as a valuable carrier for delivering ARM to the brain intranasally.

## 9. Challenges and Potential Future Directions

Despite extensive development and intensive experimental studies, the majority of drugs designed for the treatment of diseases of the central nervous system fail during clinical trials. The main reason for such a failure is a severe restriction on drug penetration into the brain imposed by the BBB. Employing the nose-to-brain delivery may potentially solve the problem. However, mucociliary clearance and the relatively low retention time of nanoparticles in the nasal cavity represent substantial challenges for the effective N2B delivery. These challenges potentially can be addressed by using mucoadhesive gels or devices with a reservoir of slow-released drugs. Such devices can be designed in a way that they (1) stick to the placatory mucosa for a long period of time that is enough to diffuse the most part of the drug into the olfactory region of the brain and (2) protect the drug from action of mucus and cilial elimination while allowing diffusion to the brain tissues. Another perspective method includes injection of a drug depo, device or hydrogel directly to the submucosal compartment of the olfactory epithelium. On such approach, known as Minimally Invasive Nasal Depot (MIND) was developed and tested [279,280]. According to this technique, a therapeutic payload is precisely delivered as a depot within the olfactory epithelium under endoscopic guidance. It is expected that the MIND technique can provide an extensive drug release to the brain up to one month after implantation.

It also should be stressed here that the N2B delivery is used most exclusively for the transport of therapeutic small molecule weight drugs. However, one perspective future direction of therapy of brain diseases (as well as other illnesses) includes the use of nucleic acids in different configurations (e.g., oligonucleotides, RNA, mRNA, etc.). Nucleic acid can be used as therapeutic moieties in several directions. They can suppress undesirable genes or mRNAs (e.g., in forms of siRNA or antisense oligonucleotides) or, in contrast, enhance the expression of therapeutic proteins or substitute defect genes. They also can be used as local or systemic vaccines to stimulate antigen presentation and therefore initiate an immune response. Similar to types of nanoparticles to those discussed above can potentially be used for the delivery of therapeutic nucleic acids alone or in combination with traditional drugs/therapeutic moieties [281]. The development and successful use of therapeutic RNA vaccines against COVID-19 viruses will hopefully stimulate pharmaceutical companies to move toward production of novel nucleic acid-based drugs, many of which were already developed and had passed pre-clinical evaluations but were rejected for being too complicated and expensive.

## 10. Conclusions

Drug delivery to the brain has been a significant challenge due to the uniqueness of BBB and the risk of adverse side effects after systemic drug delivery. Nose-to-brain delivery of therapeutic molecules can be a promising option since it can bypass BBB and increase the concentration of therapeutic molecules in the brain by direct transport via olfactory and trigeminal pathways. This approach can decrease the required drug dosage and may eventually lower the risk of systemic toxicity. Different novel nanoscale-based carriers have a potential to further escalate the drug targeting effect by delivering active molecules locally to the brain and potentially provide controlled release of therapeutics. There are some recently marketed FDA approved therapires, such as Nayzilam^®^, Valtoco^®^, or ongoing clinical trials for N2B delivery (ClinicalTrials.gov Identifier: NCT01767909, NCT03541356, NCT02503501), but most of the studied dosage forms are drug solutions (rather than nanosystems) that required oral or parenteral administration. To effectively translate the preclinical data to the clinical world, a deeper understanding of the N2B delivery system, including the pharmacokinetics and pharmacodynamics of intranasally administered active pharmaceutical ingredients and the manufacturing of a suitable device that targets the olfactory region, is necessary. Despite its challenges, N2B delivery using nanocarrier systems has great potential for the treatment of CNS disorders.

## Figures and Tables

**Table 1:** Applications of nanoparticle in N2B delivery for treatment of epilepsy.

Drug	Nanocarrier	Lipids	Surfactant/Co-Surfactant	Surface Modification	Size, nm	Z-Potential, mV	PDI	EE, %	DTE, %	DTP, %	Ref.
Letrozole	Nanoemulsion	Triacetin	Tween 80,	PEG-400	95.59 ± 2.34		0.162 ±0.012	97.37 ± 1.13	-	-	[120]
Carbamazepine	Nanoemulgel	Oleic acid	Labrasol	Xanthan gum	45–146	-	-	-	-	-	[155]
Diazepam	PLGA Nanoparticle	-	Poloxamer 407		183.2	-	-	87.8	258	61.3	[156]
Oxcarbazepine	PLGA Nanoparticle	-	Tween 80	-	256.16 ± 2.94	−15.12 ± 0.36	0.144 ± 0.024	85.1 ± 2.1	-	-	[157]
	Emulsome	Triolein	Tween 80		120.4 ± 1.45 * 101.5 ^†^	−34.1 ± 1.27 * −6.72 ^†^	0.36 * -	81.19 ± 2.3 * -	265.7	62.3	[158]
Amiloride	Nanoemulsion	Oleic acid	Tween-80/Carbitol		89.36 ± 11.18	−9.83 ± 0.12	0.231 ± 0.018	98.28 ± 0.29	1993 ± 46	586.2 ± 11.6	[159]
Thyrotropin-release hormone	PLA Nanoparticle	-	PVA	-	108 ± 12	-	-	-	-	-	[160]
Lorazepam	PLGA Nanoparticle	-	Poloxamer 407		168.2	−18.4	0.08	83.8			[161]
Lamotrigine	Liposome	Phospholipon 90G/Cholesterol	Tween 80	-	88.90 ± 1.56	-	0.247 ± 0.04	68.75 ± 0.02	-	-	[162]

Abbreviations: PDI: polydispersity index; EE: Encapsulation efficiency; DTE: Drug targeting efficiency; DTP: Direct transport percentage; PEG: polyethylene glycol; PLA: Poly (D,L-Lactic acid); PLGA: poly lactic-co-glycolic acid; PVA: polyvinyl alcohol. * Without Tween80; ^†^ With Tween 80.

**Table 2:** Applications of nanoparticles in N2B delivery for treatment of Alzheimer’s disease.

Drug	Nano-Carrier	Lipids	Surfactant/Co-Surfactant	Surface Modification	Size, nm	Z-Potential, mV	PDI	EE, %		DTE, %	DTP, %		Ref.
Tarenflurbil		PLGA Nanoparticle	-	PF-68		-	133.13 ± 7.82	−30.25 ± 2.11	0.21 ± 0.02	64.11 ± 2.21	287.24	65.18	[139]
		SLN	Glyceryl monostearate/Stearic acid/Soy lecithin	Tween 20		-	169.87 ± 10.98	−23.13 ± 2.32	0.24 ± 0.04	57.81 ± 5.32	183.15	45.41	
Donepezil		Liposome	DSPC/Cholesterol	-		PEG	102 ± 3.3	-28.31 ± 0.85	0.28 ± 0.03	84.91 ± 3.31	-	-	[99]
		Chitosan Nanosuspension	-	-		-	150–200	-	0.341	92–96	-	-	[178]
		PLGA Nanoparticle	-	PVA/Tween 80		-	89.67 ± 6.43	−36 ± 1.05	0.013 ± 0.002	88.65 ± 2.51	-	-	[179]
Galantamine		Thiolated Chitosan Nanoparticle	-	-		-	148.2–150	+27.2	0.216–0.225	87.65–89	-	-	[180]
		Liposome	SPC/Cholesterol	-		Propylene glycol	112 ± 8	−49.2 ± 0.7	-	83.6 ± 1.8	-	-	[181]
		SLN	Compritol 888 ATO	PF-127/Tween 80		-	92.0 ± 3.51	−17.22 ± 1.1	0.380 ± 0.16	83.42 ± 0.63	-	-	[182]
Memantine		Nanoemulsion	Labrasol	Tween 20/Propylene glycol			~11	-19.6	0.080	-	158.78	37.05	[183]
Pioglitazone		NLC	Capmul MCM/Tripalmitin	PF 68/Tween 80		-	211.4 ± 3.54	+14.9 ± 1.09	0.257 ± 0.108	70.18 ± 4.5	-	-	[184]
Huperzine A		PLGA Nanoparticle	-	PVA		TMC/Lactoferrin	153.2 ± 13.7	+35.6 ± 5.2	0.229 ± 0.078	73.8 ± 5.7	-	-	[185]
Rivastigmine		Chitosan Nanoparticle	-	-		-	185.4 ± 8.4	+38.4 ± 2.85	0.391 ± 0.065	85.3 ± 3.5	355 ± 13.5	71.80 ± 6.7	[186]
		Liposome	EPC/cholesterol	-		DSPE-PEG/CPP	178.9 ± 11.7	−8.6 ± 2.4	0.333 ± 0.032	30.5 ± 8.0	-	-	[187]
BACE1 SiRNA		SLN	Witepsol E 85	PVA		RVG-9R/Chitosan	335.76 ± 34.81 * 358.44 ± 25.89 ^#^	−17.31 ± 0.68 * + 10.54 ± 0.75 ^#^	0.013 ± 0.00 * 0.028 ± 0.02 ^#^	- -	-	-	[188]
R-flurbiprofen		Albumin Nanoparticle	-	-		-	284.4 ± 14.9	-	0.404 ± 0.065	-	-	-	[189]

Abbreviations: PDI: polydispersity index; EE: Encapsulation efficiency; DTE: Drug targeting efficiency; DTP: Direct transport percentage; BACE1: beta-secretase 1; DSPC: Distearoylphosphatidylcholine; DSPE: 1, 2-Distearoyl-sn-glycero-3-phosphoethanolamine; EPC: Egg phosphatidylcholine; NLC: nanostructured lipid carrier; PEG: polyethylene glycol; PF: Pluronic F; PLGA: poly lactic-co-glycolic acid; PVA: polyvinyl alcohol; RVG-9R: rabies virus glycoprotein -9 arginine; SiRNA: small interfering RNA; SLN: solid lipid nanoparticle; SPC: soya phosphatidylcholine; TMC: N-trimethyl chitosan. * without chitosan; ^#^ coated with chitosan.

**Table 3:** Applications of nanoparticle in N2B delivery for treatment of Parkinson’s disease.

Drug	Nanocarrier	Lipids	Surfactant/Co-Surfactant	Surface Modification	Size, nm	Z-Potential, mV	PDI	EE, %	DTE, %	DTP, %	Ref.
Rasagiline	Chitosan glutamate Nanoparticle	-	-	-	151.1 ± 10.31	-	0.380 ± 0.01	96.43 ± 4.23	325 ± 40	69.27 ± 2.1	[200]
Dopamine	PLGA Nanoparticle	-	PVA	PEG/Borneol/Lactoferrin	175.3 ± 9.6	−15.7 ± 0.86	0.129± 0.011	25.43 ± 5.32	-	-	[201]
Bromocriptine	Chitosan Nanoparticle	-	-	-	161.3 ± 4.7	+40.32 ± 2.78	0.44 ± 0.03	84.2 ± 3.5	633 ± 86.1	84.2 ± 1.9	[202]
Rotigotine	Chitosan Nanoparticle	-	-	-	75.37 ±3.37	+25.53 ± 0.45	0.368 ± 0.02	96.08 ± 0.01	258.10 ±17.13	53.87 ± 10.14	[203]
	PLGA Nanoparticle	-	-	PEG/Lactoferrin	122.0 ± 19.3	−21.28 ± 2.15	0.194 ± 0.023	92.57 ± 9.41	-	-	[204]
Selegiline	NLC	Stearylamine/Olive oil	PF 68 Tween 80	-	133 ± 6.08	-	0.357 ± 0.06	93 ± 5.25	-	-	[205]
	Nanoemulsion	Grape seed oil/Sefsol 218	Tween 80/Lauroglycol 90	-	61.43 ± 4.10	-34	-	-	-	-	[206]
Basic fibroblast growth factor	NLC	Gelatin	Poloxamer 188	-	172 ± 1.31	−27.6 ± 1.1	0.105 ± 0.011	86.7 ± 1.1	-	-	[91]
Geraniol/ursodeoxycholic acid conjugate	SLN	Compritol ATO 888	Span 85	-	121 ± 8.4	−22.5 ± 7.7	0.164 ± 0.03	94.5 ± 2.6	-	-	[207]
Ropinirole	SLN	Dynasan 114/Stearylamine	PF 68/Soy lecithin	-	66.22 ± 6.22	+28.19 ± 3.02	0.023 ± 0.21	61.90 ± 0.18	-	-	[208]
	Chitosan Nanoparticle	-	-	-	173.7 ± 2.32	+32.7 ± 1.5	0.39 ± 0.03	69.6 ± 3.3	-	-	[209]
	PLGA Nanoparticle	-	TPGS	-	279.4 ± 1.8	-29.4 ± 2.6	0.329 ± 0.09	72.3 ± 6.1	-	-	[210]
Pramipexole	Chitosan Nanoparticle	-	-	-	292.5 ± 8.80	+14.0 ± 2.89	0.292	91.25 ± 0.95	-	-	[211]
Naringenin	Nanoemulsion	Vitamin E Capryol 90	Tween 80/Transcutol-HP	-	38.70 ± 3.11	− 27.4 ± 0.14	0.14 ± 0.0024	-	822.71 ± 9.14	72.14 ± 5.87	[212]
Urocortin	PLGA Nanoparticle	-	Sodium cholate	OL/PEG	114.8 ± 5.6	−24.7 ± 1.5	0.193	75.5 ± 0.8	-	-	[213]

Abbreviations: PDI: polydispersity index; EE: Encapsulation efficiency; DTE: Drug targeting efficiency; DTP: Direct transport percentage; NLC: nanostructured lipid carrier; OL: odorranalectin; PDI: polydispersity index; PEG: polyethylene glycol; PF: Pluronic F; PLGA: poly lactic-co-glycolic acid; PVA: polyvinyl alcohol; SLN: solid lipid nanoparticle; TPGS: d-a-tocopheryl polyethyleneglycol 1000 succinate.

**Table 4:** Applications of nanoparticle in N2B delivery for treatment of stroke.

Drug	Nanocarrier	Surfactant/Co-Surfactant	Surface Modification	Size, nm	Z-potential, mV	PDI	EE, %	DTE, %	DTP, %	Ref.
Thymoquinone	PLGA Nanoparticle	-	Chitosan	183.5 ± 8.2	+33.63 ± 2.25	0.257 ± 0.02	73.2 ± 2.6	524.17	80.47	[225]
NR2B9c	PLGA Nanoparticle	Sodium cholate	WGA/PEG	~139	−23.3	<0.2	~50	~150	78.58	[226]
Curcumin	PNIPAM Nanoparticle	-	-	92.46 ± 2.8	-16.2 ± 1.42	0.191 ± 0.052	84.63 ± 4.2	659.23 ± 83.59	84.03 ± 1.81	[227]
Demethoxycurcumin		-	-	91.23 ± 4.2	-15.6 ± 1.33	0.183 ± 0.063	84.71 ± 3.99	667.84 ± 85.12	85.23 ± 2.19	
Bisdemethoxycurcumin		-	-	94.28 ± 1.91	−16.6 ± 1.21	0.142 ± 0.046	85.73 ± 4.31	677.12 ± 289.99	85.47 ± 2.49	

Abbreviations: PDI: polydispersity index; EE: Encapsulation efficiency; DTE: Drug targeting efficiency; DTP: Direct transport percentage; PEG: polyethylene glycol; PLGA: poly lactic-co-glycolic acid; PNIPAM: Poly-N-isopropylacrylamide; WGA: wheat germ agglutinin.

**Table 5:** Applications of nanoparticle in N2B delivery for treatment of schizophrenia.

Drug	Nanocarrier	Lipids	Surfactant/Co-Surfactant	Surface Modification	Size, nm	Z-Potential, mV	PDI	EE, %	DTE, %	DTP, %	Ref.
Quetiapine	Chitosan Nanoparticle	-	-	-	131.08 ± 7.45	+34.4 ± 1.87	0.252 ± 0.064	89.93 ± 3.85	374.93 ± 15.02	73.33 ± 4.14	[237]
	Nanoemulsion	Capmul MCM	Tween 80/Transcutol	-	144 ± 0.5	−8.131 ± 1.8	0.193 ± 0.04	91 ± 0.3	267.98 ± 3.06	63.63	[238]
Aripiprazole	PCL Nanoparticle	-	Poloxamer 188/Poloxamer 407	-	199.2 ± 5.65	−21.4 ± 4.6	-	69.2 ± 2.34	64.11 ± 4.68	74.34 ± 3.76	[239]
Olanzapine	PCL Nanoparticle	-	-	MMA/DMAEMA	254.9 ± 12.1	+22.2 ± 1.2	0.03 ± 0.01	99.00 ± 0.05	-	-	[240]
	PLGA Nanoparticle	-	Poloxamer 407	-	91.2 ± 5.2	−23.7 ± 2.1	0.120 ± 0.018	68.91 ± 2.31	-	-	[241]
Risperidone	SLN	Compritol 888 ATO	PF-127	-	148.05 ± 0.85	−25.35 ± 0.45	0.148 ± 0.028	59.65 ± 1.18	-	-	[111]
	Nanoemulsion	Capmul MCM	Tween 80/Transcutol/Propylene glycol	-	16.7 ± 1.21	−9.15 ± 2.14	0.19 ± 0.04	98.86 ± 1.21	476 ± 0.14	78 ± 1.31	[242]
Ziprasidone	Nanoemulsion	Capmul MCM	Labrasol/Transcutol	-	145.24 ± 4.75	−30.2 ± 3.21	0.186 ± 0.40	-	-	-	[243]

Abbreviations: PDI: polydispersity index; EE: Encapsulation efficiency; DTE: Drug targeting efficiency; DTP: Direct transport percentage; DMAEMA: 2-(dimethylamino) ethyl methacrylate; MMA: methyl methacrylate; NLC: nanostructured lipid carrier; PCL: polycaprolactone; PEG: polyethylene glycol; PF: Pluronic F; PLGA: poly lactic-co-glycolic acid; SLN: solid lipid nanoparticle.

**Table 6:** Applications of nanoparticle in N2B delivery for treatment of depression.

Drug	Nanocarrier	Lipids	Surfactant/Co-Surfactant	Surface Modification	Size, nm	Z-Potential, mV	PDI	EE, %	DTE, %	DTP, %	Ref.
Venlafaxine	Chitosan Nanoparticle	-	-	-	167 ± 6.5	+23.83± 1.76	0.367 ± 0.045	79.3 ± 2.6	508.59	80.34	[256]
	Alginate Nanoparticle	-	-	-	173.7 ± 2.5	+37.40 ± 1.74	0.391 ± 0.045	81.3 ± 1.9	425.77	76.52	[134]
Desvenlafaxine	PLGA Nanoparticle	-	-	Chitosan	172.5 ± 10.2	+35.63 ± 8.25	0.254 ± 0.02	76.4 ± 4.2	544.23	81.62	[257]
Buspirone	TC Nanoparticle	-	-	-	226.7 ± 2.52	-	0.483 ± 0.031	81.13 ± 2.8	78.94 ± 15.31	95.97 ± 11.31	[258]
Agomelatine	SLN	Gelucire 43/01	PVA/SDC	-	167.70 ± 0.42	−17.90 ± 2.70	0.12 ± 0.1	91.25 ± 1.7	190.02	47.37	[259]
Selegiline	TC Nanoparticle	-	-	-	215 ± 34.71	+17.06	0.214 ± 0.0421	70 ± 2.71	-	-	[131]

Abbreviations: PDI: polydispersity index; EE: Encapsulation efficiency; DTE: Drug targeting efficiency; DTP: Direct transport percentage; PEG: polyethylene glycol; PLGA: poly lactic-co-glycolic acid; PVA: polyvinyl alcohol; SDC: sodium deoxycholate; SLN: solid lipid nanoparticle; TC: thiolated-chitosan.

**Table 7:** Applications of nanoparticle in N2B delivery for treatment of other CNS diseases and pain.

Drug	Nanocarrier	Lipids	Surfactant/Co-Surfactant	Surface Modification	Size, nm	Z-Potential, mV	PDI	EE, %	DTE, %	DTP, %	Ref.
Tapentadol	Chitosan Nanoparticle	-	-	-	201.2 ± 1.5	+49.3 ± 1.2	0.201 ± 0.01	63.49 ± 1.61	321 ± 60	68.85 ± 0.49	[263]
Baclofen	PLGA Nanoparticle	-	Poloxamer 407	-	124.8	−20.4	0.225	86.45 ± 1.65	183.85	45.92	[264]
Teriflunomide	NLC	Compritol 888 ATO/Maisine 35-1/	Gelucire 44/14/Tween 20	-	99.82 ± 1.36	−22.29 ± 1.8	0.35 ± 0.01	83.39 ± 1.24	-	-	[265]
Rosmarinic acid	SLN	HSPC	Soya lecithin/Tween 80	-	149.2 ± 18.2	−38.27	0.290 ± 0.021	61.9 ± 2.2	-	-	[266]
Artemether	NLC	TM/MCT	PF 68	-	123.4 ± 3.6	−34.4 ± 1.2	-	91.2 ± 2.5	278.16	64.02	[267]
Leucine-enkephalin	TMC Nanoparticle	-	-	-	443 ± 23	+15 ± 2	0.317 ± 0.17	78.28 ±3.8	-	-	[130]
Cyclobenzaprine	TC Nanoparticle	-	SDC	-	282.9 ± 15.6	+27.7 ± 1.2	-	80.20 ± 3.2	2101	95.24	[132]
Lamotrigine	PLGA Nanoparticle	-	Poloxamer 407	-	184.6	−18.8	0.082	84.87 ± 1.2	129.81	22.96	[268]
Ondansetron	SLN	Glycerol monostearate	Lecithin/Poloxamer 188	-	299.67	−16.5	0.296	49.82	-	-	[269]

Abbreviations: PDI: polydispersity index; EE: Encapsulation efficiency; DTE: Drug targeting efficiency; DTP: Direct transport percentage; HSPC: Hydrogenated soya phosphatidyl choline; MCT: medium chain triglyceride; NLC: nanostructured lipid carrier; PDI: polydispersity index; PF pluronic F; PLGA: poly lactic-co-glycolic acid; SDC: sodium deoxycholate; SLN: solid lipid nanoparticle; TC: thiolated-chitosan; TM: trimyristin; TMC: trimethyl chitosan.

## Data Availability

Not applicable.
